# Acid resistance system CadBA is implicated in acid tolerance and biofilm formation and is identified as a new virulence factor of *Edwardsiella tarda*

**DOI:** 10.1186/s13567-021-00987-x

**Published:** 2021-09-14

**Authors:** Chunmei Du, Xiaoping Huo, Hanjie Gu, Dongmei Wu, Yonghua Hu

**Affiliations:** 1 College of Basic Medicine, Jiamusi University, 154007 Jiamusi, China; 2 Institute of Tropical Bioscience and Biotechnology, Hainan Academy of Tropical Agricultural Resource, CATAS, 571101 Haikou, China; 3 Laboratory for Marine Biology and Biotechnology, Pilot National Laboratory for Marine Science and Technology (Qingdao), 266071 Qingdao, China; 4 College of Life Science, Jiamusi University, 154007 Jiamusi, China; 5 Heilongjiang Provincial Key Laboratory of New Drug Development and Evaluation of the Efficacy of Toxicology, 154007 Jiamusi, China; 6 Hainan Provincial Key Laboratory for Functional Components Research and Utilization of Marine Bio-Resources, 571101 Haikou, China

**Keywords:** *Edwardsiella tarda*, acid resistance, *cadBA*, biofilm, pathogenicity, regulation

## Abstract

*Edwardsiella tarda* is a facultative intracellular pathogen in humans and animals. The Gram-negative bacterium is widely considered a potentially important bacterial pathogen. Adaptation to acid stress is important for the transmission of intestinal microbes, so the acid-resistance (AR) system is essential. However, the AR systems of *E. tarda* are totally unknown. In this study, a lysine-dependent acid resistance (LDAR) system in *E. tarda*, CadBA, was characterized and identified. CadB is a membrane protein and shares high homology with the lysine/cadaverine antiporter. CadA contains a PLP-binding core domain and a pyridoxal phosphate-binding motif. It shares high homology with lysine decarboxylase. *cadB* and *cadA* are co-transcribed under one operon. To study the function of the *cadBA* operon, isogenic *cadA*, *cadB* and *cadBA* deletion mutant strains TX01Δ*cadA*, TX01Δ*cadB* and TX01Δ*cadBA* were constructed. When cultured under normal conditions, the wild type strain and three mutants exhibited the same growth performance. However, when cultured under acid conditions, the growth of three mutants, especially TX01Δ*cadA*, were obviously retarded, compared to the wild strain TX01, which indicates the important involvement of the *cadBA* operon in acid resistance. The deletion of *cadB* or *cadA*, especially *cadBA*, significantly attenuated bacterial activity of lysine decarboxylase, suggesting the vital participation of *cadBA* operon in lysine metabolism, which is closely related to acid resistance. The mutations of *cadBA* operon enhanced bacterial biofilm formation, especially under acid conditions. The deletions of the *cadBA* operon reduced bacterial adhesion and invasion to Hela cells. Consistently, the deficiency of *cadBA* operon abated bacterial survival and replication in macrophages, and decreased bacterial dissemination in fish tissues. Our results also show that the expression of *cadBA* operon and regulator *cadC* were up-regulated upon acid stress, and CadC rigorously regulated the expression of *cadBA* operon, especially under acid conditions. These findings demonstrate that the AR CadBA system was a requisite for the resistance of *E. tarda* against acid stress, and played a critical role in bacterial infection of host cells and in host tissues. This is the first study about the acid resistance system of *E. tarda* and provides new insights into the acid-resistance mechanism and pathogenesis of *E. tarda*.

**Supplementary Information:**

The online version contains supplementary material available at 10.1186/s13567-021-00987-x.

## Introduction

*Edwardsiella* was isolated from infected humans and animals and identified as a new genus of Enterobacteriaceae in 1965 [1]. Recently, the *Edwardsiella* genus was classified into five species, including *E. tarda*, *E. anguillarum*, *E. ictaluri*, *E. hoshinae*, and *E. piscicida* [2, 3]. *E. tarda* (now is also considered to be *E. piscicida*), a member of the family Enterobacteriaceae, is a gram-negative, motile, facultative anaerobe and rod-shaped bacterium [4, 5]. *E. tarda* has broad host ranges including aquatic animals, reptiles, amphibians, birds, mammals and humans [6–8], therefore being a significant zoonotic pathogen [9]. This pathogen can cause serious systemic infections and high mortality in both seawater and freshwater fish, accounting for severe economic losses and heavily influencing the healthy development of aquaculture [10, 11]. Meanwhile, *E. tarda* is also a fatal gastro-/extraintestinal pathogen in humans. The most frequent manifestation of gastrointestinal infection is a *Salmonella*-like gastroenteritis. The extraintestinal manifestations of *E. tarda* infection include meningitis, cholecystitis, endocarditis, peritonitis, bacteraemia, septicemia, empyema, liver abscess, intra-abdominal abscess, tubo-ovarian abscess, and skin and soft tissue infection [12, 13], which can eventually lead to severe, systemic, and life-threatening infections. Consequently, *E. tarda* is widely considered a potentially important bacterial pathogen. 

Like other enteric pathogens within the same family such as *Escherichia coli*, *Shigella*, and *Salmonella* species, *E. tarda* possesses a lot of virulence systems including type III and type VI secretion systems, quorum sensing, two-component systems, and exoenzymes to gain entry into and to survive within the host [3]. Before causing diseases in hosts, pathogenic bacteria often encounter varying environmental stresses including temperature, osmolarity, oxidative stress, pH fluctuation, famine and so on. Among these environmental stresses, pH shifts are the most frequent dilemma for pathogens [14, 15]. These acidic environments include acid soil and fermented food, the harsh inorganic and organic volatile fatty environment in the gastrointestinal tract, as well as mild to moderate acids in phagosomes of cell macrophages [16]. Therefore, an important strategy for pathogens is to adapt to the acidic stress environment of the host. To counter intracellular and extracellular acid stresses, bacteria have developed passive and active protective mechanisms [17]. Among these mechanisms, the amino acid-dependent acid resistance (AR) system is composed of a cytoplasmic decarboxylase that catalyzes a proton-dependent decarboxylation of a substrate amino acid, and an inner membrane substrate/product antiporter that exchanges external substrate for internal product [18]. In *E. coli*, the AR systems that have been identified are the glutamic acid-dependent acid resistance (GDAR) system [19], the arginine-dependent acid resistance (ADAR) system [20], the lysine-dependent acid resistance (LDAR) system [21] and the ornithine-dependent acid resistance (ODAR) system [22]. Each of these systems has a different optimum pH and is capable of providing protection against acid stress over a broad range of pH values [23].

The LDAR system is a mild acid tolerance response system. In *E. coli*, the LDAR system consists of the cytoplasmic protein CadA, the integral membrane proteins CadB, and one-component regulator CadC [24]. CadA, the inducible lysine decarboxylase, is encoded by *cadA* gene that converts lysine to cadaverine with consumption of a proton [25, 26]. CadB, the lysine/cadaverine antiporter with 12 transmembrane helices, is encoded by the *cadB* gene that transports lysine into the cell and exports cadaverine. *cadB* and *cadA* are organized in one operon which work together to maintain pH homeostasis inside the cell and extracellular environment [27]. CadC is a membrane-integrated transcription activator located upstream of *cadB*. The regulator belongs to the ToxR family, consisting of a cytoplasmic N-terminal DNA-binding domain that regulates transcription, a transmembrane helix, and a C-terminal periplasmic sensory domain [28]. Under conditions of acidic pH and exogenous lysine, CadC interaction with the lysine-specific permease LysP senses signal transduction to the DNA-binding domain via the transmembrane helix [29]. DNA-binding domain undergoes structural changes that enable the winged protein to interact with the target promoter of cadBA and activated expression of the *cadBA* operon [30]. The LDAR system has been thoroughly studied in Enterobacteria such as *E. coli* [31], *Salmonella typhimurium* [15] and *Vibrio* [32] because of direct links between the efficiency of the acid stress response and pathogenicity, but little is known about biological roles in *E*. *tarda*. 

In this study, we investigated the properties of *cadBA* operon in *E. tarda*, and analyzed its distinct roles in the physiological fitness and pathogenesis in in vitro and in vivo models of infection with single and double deletion mutations in *cadB* and *cadA*. Our results uncover a vital role of *cadBA* operon in *E. tarda* survival under acidic stress environment and in host cells, and provide the first insights into the pathogenicity of the AR system in *E. tarda*.

## Materials and methods

### Strains and culture conditions

*E. coli* DH5ɑ was purchased from TransGen (Beijing, China) and used for genetic manipulation. *E. coli* SM10 λpir or D1000 was purchased from Biomedal (Sevilla, Spain) and used for preparation of suicide vectors. *E. tarda* TX01 was isolated from diseased fish [33]. Bacteria were grown in Luria–Bertani (LB) broth or on LB agar at 37 °C (for *E. coli*) or 28 °C (for *E. tarda*). When required, antibiotics were added to the media as follows: ampicillin (Amp), 100 μg/mL; chloramphenicol (Cm), 30 μg/mL; polymyxin B (poly B) or colistin (Col), 100 μg/mL; tetracycline (Tc), 20 μg/mL and kanamycin (Km), 50 μg/mL, respectively.

### Total RNA isolation, cDNA synthesis, and co-transcriptional verification

The experiment was performed as previously reported [34]. Overnight cultures of *E. tarda* TX01 were grown in LB broth at 28 °C and used for RT-PCR. Total RNA was isolated using the HP Total RNA kit (Omega Bio-Tek, USA) according to the manufacturer’s instructions. cDNA synthesis was performed with Superscript II reverse transcriptase (Invitrogen, USA). The *cadBA* co-transcriptional fragment was located at the 3ʹ end of *cadB* and 5ʹ end of *cadA*, and amplified with specific primers CadBAF and CadBAR using total RNA, cDNA, and genomic DNA as the template, respectively. The sequences of primers used are shown in Table 1.

**Table 1:** **Oligonucleotide primers used in this study**

Primer name	Sequence (5′-3′)
CadAKOF1	GGATCCCGCATTTTGCCTGGAGA (*BamH*I)
CadAKOR1	TTAGCCACGCCGTAGAAATCATAGAACA
CadAKOF2	TCTACGGCGTGGCTAAATACCTGGACG
CadAKOR2	GGATCCGGGTAGTGAGCACCGATTT (*BamH*I)
CadAKOF3	TCAGCGAGATGAACGAGCA
CadAKOR3	TGACCACGCAGTTCCATCT
CadBKOF1	GGATCCTATTGCGACACGCTGAG (*BamH*I)
CadBKOR1	CGGAAGCCGAAGAAGGTGGACAGGTA
CadBKOF2	CCTTCTTCGGCTTCCGTGAAAATGA
CadBKOR2	GGATCCCGAATGGGTTCTTCTTTGA (*BamH*I)
CadBKOF3	GAGGTTGGTCCTGCTTTTG
CadBKOR3	GAAGCGAATCAGGTCAACG
CadBAPF1	GATATCATTTGTATAATGATTGACCAC (*EcoR*V)
CadBAPR1	GATATCGGTCAAGTTGCTCCTGATT (*EcoR*V)
CadBAPR2	ATATTCATGGTCAAGTTGCTCCTGATT
CadAF1	ACTTGACCATGAATATTATTGCCATCCTG
CadAR1	GATATCTTACTTCTGTTCAGTTTTGAGC (*EcoR*V)
CadBR1	GATATCTTATGCCGCGGCGTTACT (*EcoR*V)
CadBAF	CCCGAAAGTTTACGGTGAGA
CadBAR	GCAGGTGCTCGTTCATCTCG
CadCRTF	GGTACTTGAGCCCAGACTCATCGA
CadCRTR	GCACGGTCACGATATACTCTGGC
CadBRTF	GCGCCATGTCACTGGCCTATGT
CadBRTR	TTCAGCACCGAAGAAGGTGGACAG
CadARTF	GCACGTCTGTGCGGCGTTATCT
CadARTR	TTCAGCGCGATGTCTTCGGCTG
CadBARTF	GAACAACAGTAACGCCGCGGCAT
CadBARTR	CCCATGTGATTCAGGATGGCAATAATATTC
3006RTF	CTCGCCGACCTTCAAGCGGATT
3006RTR	CAGCATTTCGTTCAGCGCCGTGAT
1884RTF	CTTGGTCGGTGCAATGTCGCTG
1884RTR	GGAAGAAGACCGACAGATAGGCG

### The expression of acid resistance genes under acidic conditions and in *cadC* mutant

To study expression of acid resistance genes under exposure to acid stress conditions, the exponential phase cultures of TX01 were treated for 1 h in LB media supplemented with 5 mM l-lysine (pH 5.5) in static cultivation. Total RNA was extracted with an HP Total RNA kit (Omega Bio-Tek, USA) according to the manufacturer’s instructions. cDNA synthesis was performed as described above. RT-qPCR was carried out as reported previously [35]. The relative transcriptional level of acidic resistance genes (*cadC*, *cadB*, *cadA*, and *cadBA*) in *E. tarda* were determined using the 2^−△△CT^ method with 16S rRNA used as a reference gene.

### Constructions of mutant strains TX01Δ*cadA*, TX01Δ*cadB*, and TX01Δ*cadBA* and plasmid-complemented strains TX01Δ*cadAC *and TX01Δ*cadBC*

The constructions of mutants TX01Δ*cadA*, TX01Δ*cadB*, and TX01Δ*cadBA* and complementary strains TX01Δ*cadA*C and TX01Δ*cadB*C were performed as reported previously [35]. The primers used in this study are listed in Table 1. Primers CadAKOF1/CadAKOR1 and CadAKOF2/CadAKOR2 were used for the construction of TX01Δ*cadA* mutant, in which a 1023 bp segment (517 to 1539 bp in the ORF) in-frame deletion was created. Primers CadBKOF1/CadBKOR1 and CadBKOF2/CadBKOR2 were used for the construction of TX01Δ*cadB* mutant, in which a 636 bp segment (337 to 972 bp in the ORF) in-frame deletion was created. The transconjugants were selected on LB agar plates supplemented with 10% sucrose. *cadA* single-knockout strain was named as TX01Δ*cadA*, and *cadB* single-knockout strain was named as TX01Δ*cadB*. A similar operation was carried out to construct the double-knockout strain TX01Δ*cadBA*.

For construction of TX01Δ*cadA* complementary strain, a 2145-bp *cadA* coding region and 418-bp putative promoter region were cloned with primers CadAF1/CadAR1 and CadBAPF1/CadBAPR2, respectively. Two DNA fragments were fused together by overlapping PCR with CadBAPF1/CadAR1 and verified by sequencing. The fused PCR fragment was digested with *EcoR*V, ligated into plasmid pJR21 digested with *Pme*I. The resulting plasmid was transformed into TX01Δ*cadA* by conjugation transfer. The complementary strain TX01Δ*cadA*C was screened on LB agar plates supplemented with 100 μg/mL polymyxin B and 20 μg/mL tetracycline, and further confirmed by PCR. For construction of TX01Δ*cadB* complementary strain, a 1332-bp *cadB* coding region and a 418-bp upstream of transcriptional start site was amplified from TX01 with primers CadBAPF1/CadBR1. The following experimental operations are similar to the construction of TX01Δ*cadA*C. The complementary strains were named as TX01Δ*cadA*C and TX01Δ*cadB*C, respectively.

### Resistance to environmental stresses

The overnight cultures of TX01, TX01Δ*cadA*, TX01Δ*cadB* and TX01Δ*cadBA* were grown in LB media, diluted to a final concentration of about 10^5^ CFU/mL. For acid stress, aliquots of (200 μL) cultures in LB broth at pH gradients (from 7.0 to 4.5) were distributed into the wells of Bioscreen C plate. For the iron deficiency assay, bacteria were added into fresh LB media with 50 μM of 2, 2ʹ-dipyridyl (Dp). For oxidation stress assay, bacteria were added into fresh LB media with 500 μM of diamide. Growth curves were monitored at 2-h intervals through the measurement of the absorbance at 600 nm using Bioscreen C Automated Growth Curve System (OyGrowth Curves Ab Ltd, Finland).

### Lysine decarboxylase assay

Lysine decarboxylase broth was purchased from Hopebio (Qingdao, China) and used for the lysine decarboxylase (LDC) qualitative measurement. Various overnight cultures of bacteria (TX01, TX01∆*cadA*, TX01∆*cadB*, TX01∆*cadBA*, TX01∆*cadA*C and TX01∆*cadB*C) were diluted to an optical density of 0.5 at 600 nm. A 50-μL aliquot of the cultures were inoculated into lysine decarboxylase broth after being washed two times and resuspended with sterile water. Sterile paraffin-coated liquid (300 μL) was then added into inoculated media to create an anaerobic environment and the cultures were incubated at 28 °C for 10, 20 and 30 h. Lysine decarboxylase broth without l-lysine was taken as a blank control. A positive test is indicated if the control tube is yellow and the LDC-positive tube is purple or red brown color. On the contrary, a negative test is indicated if both the control tube and the LDC-positive tube are yellow.

Strains were grown in LB broth at 28 °C until OD_600_ of 0.5. Culture aliquots were collected and normalized to an OD_600_ of 1.0. Quantification of lysine decarboxylase activity was then carried out as described previously [36]. Lysine decarboxylase activity is a measure of lysine converted to cadaverine per time (min) per unit cell density, and was determined by *A*_340_ between the sample incubated with or without lysine using the following equation:

$$[A340/\left( {{\text{time}}\, \times \,{\text{OD}}_{600} } \right)]\, \times \,1000.$$

### Biofilm assay

Biofilm formation was assayed using crystal violet staining as previously described [37]. Overnight cultures of TX01, TX01Δ*cadA*, TX01Δ*cadB*, TX01Δ*cadBA*, TX01Δ*cadA*C and TX01Δ*cadB*C were grown in LB media, diluted to 10^5^ CFU/mL, and then resuspended in LB media at pH 7.0 and 5.5. 200 μL of cultures were transferred into 96-well polystyrene plates and incubated at 28 °C for 24 h under static conditions. The media and planktonic cells were discarded after culture and the wells were washed three times with PBS. The adhered cells were treated with Bouin fixative (Solarbio, China) for 1 h and then stained with 1% crystal violet (CV, Sigma) solution for 20 min, followed by the removal of unbound crystal violet by washing several times with PBS. The CV bound to biofilm was solubilized with 200 μL methanol and absorbance was measured at 570 nm.

For confocal laser scanning microscopy (CLSM) observation, TX01, TX01Δ*cadA*, TX01Δ*cadB* and TX01Δ*cadBA* were cultured in LB broth on glass-bottom dishes at 28 °C for 24 h. Non-adherent cells and culture fluid were removed and washed three times with PBS. The biofilms were stained with a LIVE/DEAD BacLight bacterial viability kit L-13152 (Invitrogen-Molecular Probes, USA) for 15 min at room temperature in dark conditions. The fluorescent images were acquired using a FV1000 confocal laser scanning microscope (Olympus, Japan).

### Motility assays

Swimming motility assays were performed using swimming plates which contain LB media with 0.3% (W/V) agar at pH 7.0 or pH 5.5. Overnight cultures of TX01, TX01Δ*cadA*, TX01Δ*cadB*, TX01Δ*cadBA*, TX01Δ*cadA*C and TX01Δ*cadB*C were cultured in LB media to an OD_600_ of 0.5. Aliquots of (1 μL) cell suspensions were inoculated into swimming plates by submerging pipette tips in the cultures and pricking the center of the plates. Plates were then incubated for 18 h, and the swimming motility zone was measured.

### Invasion of eukaryotic cell lines

The human cervical epithelial cell line HeLa and the murine monocyte-macrophage cell line RAW264.7 were cultured in Dulbecco Modified Eagle medium (DMEM) supplemented with 10% (V/V) fetal bovine serum (FBS, Sigma), 100 U/mL penicillin G (Sigma), and 100 μg/mL streptomycin (Sigma) at 37 °C with 5% CO_2_.

The interaction of *E*. *tarda* and host cells was studied as reported previously [38]. Briefly, *E. tarda* TX01, TX01Δ*cadA*, TX01Δ*cadB*, TX01Δ*cadBA*, TX01Δ*cadA*C, and TX01Δ*cadB*C strains were grown in LB broth at 30 °C and then transferred into DMEM without shaking until the optical density at 600 nm reached 1.0. HeLa cells were seeded and cultured in 96-well cell culture plates, and infected with 100 μL of *E. tarda* at a multiplicity of infection (MOI) of 10:1. To detect bacterial adhesion, after incubation at 28 °C for 1 and 2 h, the monolayers were washed three times with PBS and lysed with 200 μL of 1% (V/V) Triton X-100 for 10 min; series diluted bacteria were quantified by LB agar plates supplemented with polymyxin B.

Bacterial replication in RAW264.7 cells was performed as previously [39]. Briefly, RAW264.7 cells were infected with 100 μL *E. tarda* strains at MOI of 10:1. The monolayers were washed with PBS and then in cultured DMEM containing 200 μg/mL of gentamicin for 2 h to kill the extracellular bacteria, then they were maintained in DMEM including 10 μg/mL of gentamicin for 1 h. Subsequently, the cultures were incubated at 28 °C for 0, 2, 4, and 8 h. The monolayers were washed three times with PBS and lysed with 1% (V/V) Triton X-100 for enumeration.

For microscopy observation, *E. tarda* was transformed with plasmid pGFP_UV_ expressing green fluorescence protein by electroporation using Gemini Twin Wave Electroporators [40]. RAW264.7 cells were cultured in glass-bottom dishes and infected with TX01, TX01Δ*cadA*, TX01Δ*cadB* and TX01Δ*cadBA* strains possessing the pGFP_UV_ plasmid at 30 °C for 0, 2, and 4 h. The cells were washed three times with PBS and fixed by polyformaldehyde for 30 min, and then labeled with DAPI (Solarbio, China). The cells were observed by a confocal microscope (Olympus Fluoview FV1000, Japan) after washing three times with PBS.

### Fish and experimental challenges for bacterial dissemination in vivo

Healthy Tilapias (average weight 13.0 g) were purchased from a commercial fish farm in Haikou, and fed in aerated water at 25 ± 1 °C for 2 weeks. Before the experiment, the fish were randomly sampled and checked for the presence of bacteria in the blood, liver, kidney and spleen. For tissue dissemination analysis, TX01, TX01Δ*cadA*, TX01Δ*cadB*, TX01Δ*cadBA*, TX01Δ*cadA*C, and TX01Δ*cadB*C were cultured in LB broth to an OD_600_ of 0.5. The cells were washed with PBS and resuspended in PBS to 10^7^ CFU/mL. Tilapia were divided randomly into six groups and infected by intramuscular (i. m.) injection with 50 μL of various bacterial suspensions, respectively. Fish were euthanized with an overdose of MS222 (tricaine methanesulfonate) (Sigma, USA) at 24 and 48 h post-infection. Subsequently the spleen and kidney of the fish were taken aseptically, and the recoveries of bacteria in the tissues were determined as previously [39]. The assay was performed in triplicate.

### Transcriptional regulation of *cadBA* by CadC

In the study of *cadC*, we obtained *cadC* mutant TX01Δ*cadC*. The expression of *cadBA* operon in TX01 and TX01Δ*cadC* at acid condition was examined by RT-qPCR. To examine *cadBA* expression in in vitro conditions, the speculative promoter of *cadBA* (the 418 bp DNA upstream of *cadBA* operon), P418, was cloned with primers CadBAPF1/CadBAPR1 and was inserted into the *BamH*I site of pSC11, a promoter probe plasmid [41], which resulted in pSC418. pSC418 and pJR21C which expressed the CadC were introduced into *E. coli* DH5α by co-transformation, and cultured on X-gal (5-bromo-4-chloro-3-indolyl-beta-d-galactopyranoside) plate. Meanwhile, pJR21 was used as the control. To study the transcriptional regulation of P418 by CadC in vivo, the amplified P418 fragment was inserted into the *Sma*I site of p181-lux, a promoter probe plasmid based on pACYC177 and pGL28. The resulting plasmid was digested with *Smi*I and the fragment that included P418 and luciferase gene was inserted into the *Pme*I site of pJR21, resulting in pJR21-418-lx. pJR21-418-lx was introduced into TX01 and TX01Δ*cadC* which is the *cadC* mutant by electro-transformation, respectively. The transformants were screened on LB agar plates supplemented with 100 μg/mL polymyxin B and 20 μg/mL tetracycline. The log-phase transformants were cultured in normal conditions (pH = 7.0) or in LB broth (pH = 5.5) with 5 mM l-lysine for 1 h, and subjected to luciferase assay using the Firefly Luciferase Reporter Gene Assay Kit (Beyotime, China).

### Statistical analysis

All data were analyzed statistically using the analysis of variance (ANOVA) with SPSS 18.0 software (SPSS Inc., Chicago, IL, USA), and *P* < 0.05 was considered statistically significant. Each experiment was performed three times, data are presented as the means ± SEM (*N* = 3). *N*, the number of times the experiment was performed. Statistical significance (*, *P* < 0.05; **, *P* < 0.01) was obtained using an ANOVA test with SPSS 18.0 software (SPSS Inc., Chicago, IL, USA).

## Results

### Characterization of *cadBA* operon sequence and its co-transcription verification

Although some genes or factors were identified to contribute to acid resistance of *E. tarda*, there is not any report on the classical acid resistance systems in *E. tarda*. In order to further explore *E. tarda*’s mechanism of acid resistance, a lysine-dependent acid resistance (LDAR) system, CadBA, was identified and characterized in this study. The *cadB* of *E. tarda* (ETAE_0756) consists of 1332 bp ORF that encodes a lysine/cadaverine antiporter composed of 443 amino acids with a calculated molecular mass of 46.1 kDa and a theoretical pI of 9.01. Structural analysis suggested that CadB is a membrane protein with 12 transmembrane helices. Multiple sequence alignment shows that CadB shares high overall amino acid sequence identities (66–84%) with lysine/cadaverine antiporter homologues of *Salmonella enterica*, *Klebsiella pneumoniae*, *E. coli*, *Shigella flexneri*, *Vibrio cholerae*, and *Aeromonas veronii* (Additional file 1).

The *cadA* of *E. tarda* (ETAE_0757) consists of 2145 bp ORF that encodes a lysine decarboxylase composed of 714 amino acids with a calculated molecular mass of 81.0 kDa and a theoretical pI of 5.53. CadA includes an N-terminal domain (residues 14 to 124), a PLP-binding core domain (residues 131 to 441), and a C-terminal domain (residues 571 to 695), which contains a pyridoxal phosphate binding motif formed by 15 residues. Multiple sequence alignment showed that CadA shares high overall amino acid sequence identities (70–86%) with lysine decarboxylase homologues of *E. coli*, *Salamae*, *A. veronii*, *V. cholerae*, *K. pneumoniae*, and *Shigella boydii* (Additional file 1).

**Figure 1:**
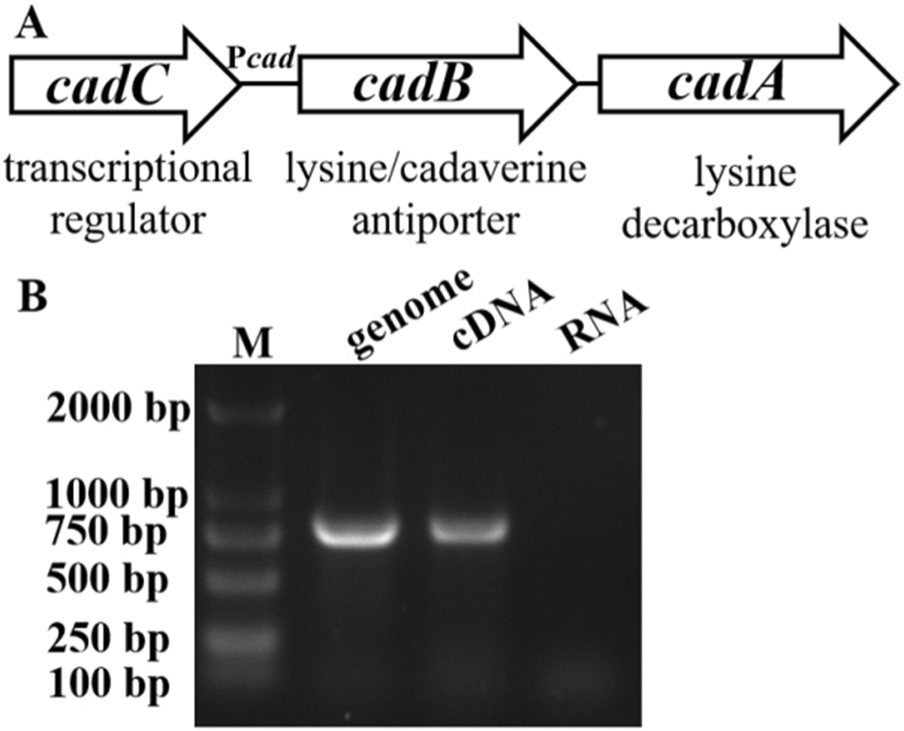
**Schematic organization and co-transcriptional verification of the *cadBA* operon in *E*. *tarda*.A** Schematic organization of the *cadBA* operon in *E*. *tarda*. The *cadBA* operon consists of *cadB* and *cadA*. The *cadBA* operon is under the control of the predictive promoter P*cad.* The upstream gene of the *cadBA* operon is *cadC*, which encodes transcriptional regulator. Therefore, the operon was named *cadBA*. **B** Verification of *cadB* and *cadA* co-transcription. Genomic DNA and total RNA were isolated from overnight cultures of *E. tarda*. RNA was treated with DNase I and cDNA was synthesized. PCR were conducted with specific primer pair CadBAF/CadBAR using genomic DNA, cDNA, and RNA as the template. PCR products were analyzed by agarose gel electrophoresis.

The *cadB* and *cadA* of numerous bacteria such as *E. coli*, *V. cholerae*, *S*. *typhimurium* are co-transcribed under one operon, providing advantages to counter low intracellular and extracellular pH. In *E. tarda*, there is a 148 bp intergenic spacer region between *cadB* and *cadA* (Figure 1A). To verify whether the two genes are co-transcribed, a pair of primers annealing to the 3′ end of *cadB* and 5′ end of *cadA* was designed. PCR was performed using total RNA, complementary DNA (cDNA), and genomic DNA (gDNA) of *E. tarda* as templates, respectively. The results show that the predicted PCR product of 795 bp was amplified in the reaction using cDNA and gDNA, but not in RNA (Figure 1B), which indicates that *cadB* and *cadA* are co-transcribed under one operon. The operon is named as *cadBA*. The upstream gene of the *cadBA* operon is a *cadC* encoded transcriptional regulator, and there is a predictive promoter between *cadB* and *cadC*, P*cad* (Figure 1A).

### Constructions of mutant strains TX01Δ*cadA*, TX01Δ*cadB*, and TX01Δ*cadBA* and complementary strains TX01Δ*cadAC*, and TX01Δc*adBC*

To verify the function of the *cadBA* operon, the *cadA* and *cadB* genes were knocked out by markerless in-frame deletion of region encoding amino acid residues 173 to 513 and 113 to 324, respectively. Meanwhile, the whole operon was also knocked out by markerless in-frame deletion of the region from the N-terminal 113 amino acid residues of CadB to C-terminal 202 amino acid residues of CadA. Deletion of the genes were verified by PCR with specific primers and sequencing (Additional file 2). The resulting mutants were named TX01Δ*cadA*, TX01Δ*cadB*, and TX01Δ*cadBA.* The genetic complementation of TX01Δ*cadA* and TX01Δ*cadB*, TX01Δ*cadA*C and TX01Δ*cadB*C, were also obtained.

### *cadBA* is involved in adversity resistance

**Figure 2:**
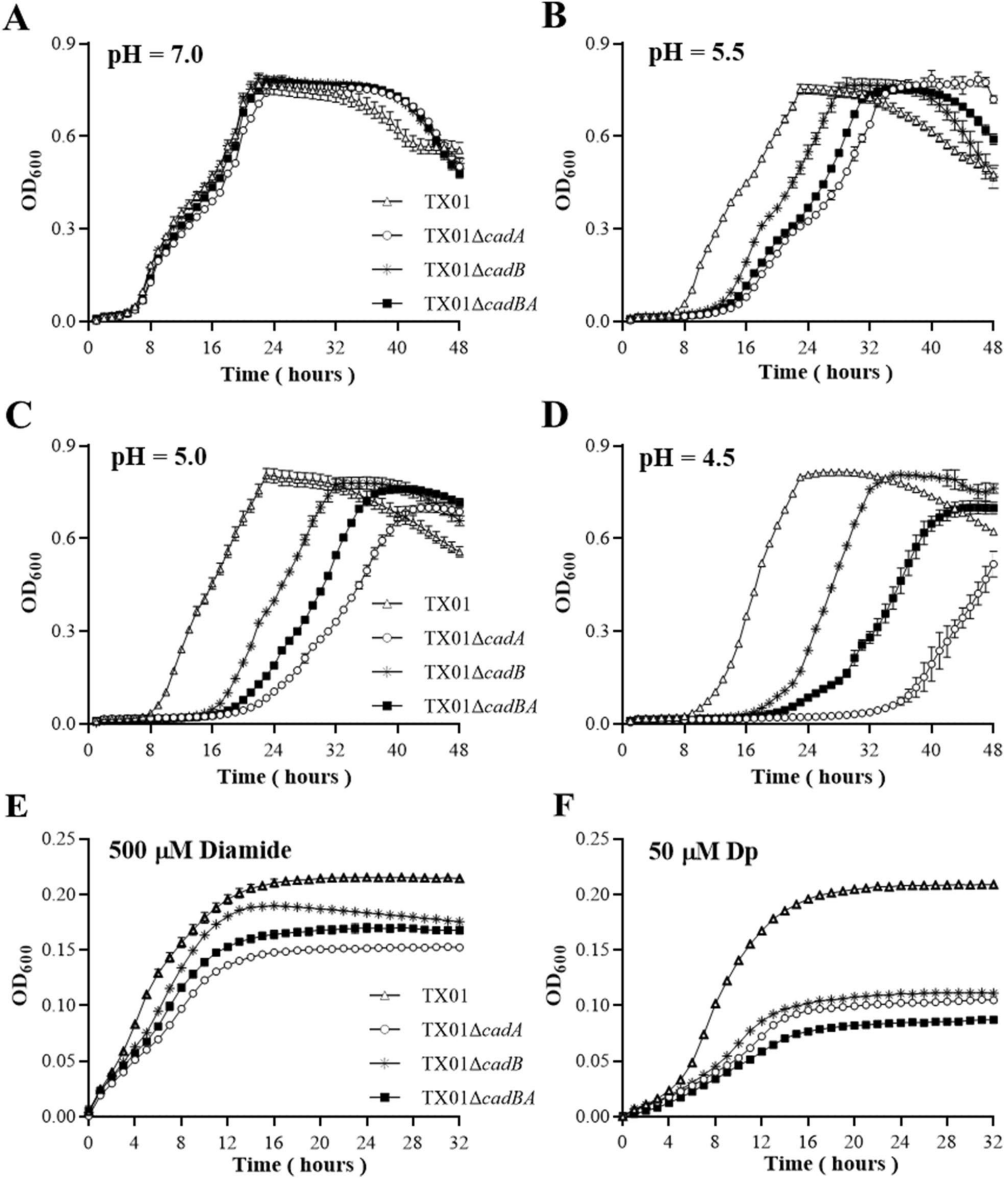
**Growth analysis of *E. tarda* in different conditions.** The wild-type TX01, its isogenic *cadA*, *cadB* and *cadBA* deletion mutant strains TX01Δ*cadA*, TX01Δ*cadB*, and TX01Δ*cadBA* were cultured to the exponential phase. After diluting serially, bacteria were cultured in fresh LB broth with pH adjusted to 7.0, 5.5, 5.0, and 4.5 (**A**–**D**). Bacteria were added into fresh medium with 500 μM of diamide (**E**) and 50 μM of 2,2ʹ-dipyridyl (Dp) (**F**), respectively. Cell density was monitored at 2-h intervals by measuring the OD_600_. Experiment was performed three times, data are presented as the means ± SEM (*N* = 3). N, the number of times the experiment was performed.

In the process of infecting the host or host cell, pathogens inevitably face some environmental pressures, such as acid stress, iron deficiency, and oxidative stress. Previous studies indicated that *cadBA* operon in several bacteria were essential for physiological fitness to acid stress. We want to know whether *cadBA* in *E. tarda* has any effects on bacterial resistance to acid stress and some other environmental pressures. For this purpose, wild type strain TX01, *cadBA* operon deletion mutant strains TX01Δ*cadA*, TX01Δ*cadB*, and TX01Δ*cadBA* were cultured in LB broth with a pH range of 7.0 to 4.5. No difference was observed between the growth characteristics of the wild-type and three mutants under normal conditions (pH = 7.0) (Figure 2A). When exposed to the acidic environment (pH = 5.5 to 4.5), the growths of four strains were retarded to varying degrees. Compared to wild type strain TX01, three mutants were obviously retarded. TX01Δ*cadA* was most affected by acid stress, followed by TX01Δ*cadBA* and TX01Δ*cadB*, but the cell densities of three mutants were similar to that of TX01 at the stationary phase (Figures 2B–D). When the pH of the medium adjusted to pH 4.0, mutants failed to grow (data not shown).

When grown in LB medium containing 500 μM of oxidant diamide, the growths of four strains were retarded and three mutants displayed slightly slower growth rates than TX01 (Figure 2E). When grown in LB medium containing 50 μM of dipyridyl (a kind of iron chelator), the growths of four strains were retarded and three mutants displayed obviously slower growth rates than TX01, but there was no obvious difference of growth amongst three mutants (Figure 2F). These results suggest that the deletion of *cadA* or *cadB* does not impair the growth of *E. tarda* under normal conditions, but to a certain extent, affects bacterial growth under iron deficiency and oxidative stress, especially under acid stress.

### *cadBA* participates in lysine metabolism

**Figure 3:**
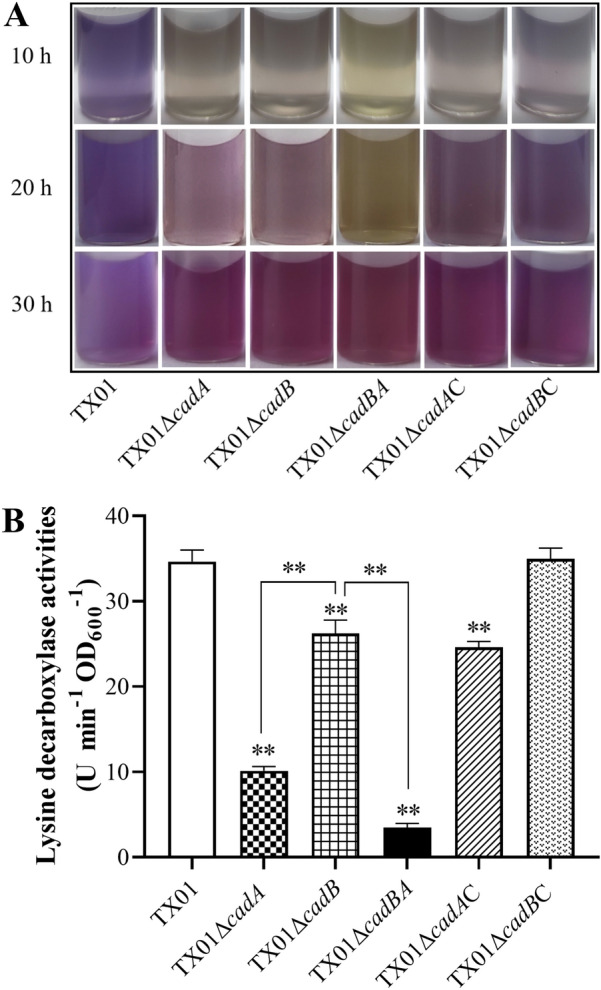
**The activities of lysine decarboxylase in *E*. *tarda*.A** TX01, TX01Δ*cadA*, TX01Δ*cadB*, TX01Δ*cadBA*, TX01Δ*cadA*C, and TX01Δ*cadB*C were cultured in lysine decarboxylase broth with lysine in static condition at 28 ℃ for 10, 20 and 30 h, respectively. A light purple indicates a weak positive result. Purple and yellow indicate the presence and absence of LDC, respectively. **B** The same strains were cultured in LB broth to an OD_600_ of 0.5 and normalized to an OD_600_ of 1.0, buffered at pH 6.8. The activities of lysine decarboxylase were determined as described in the text. The experiment was performed three times, data are presented as the means ± SEM (*N* = 3). N, the number of times the experiment was performed. **, *P* < 0.01.

The inducible lysine decarboxylase can convert lysine to cadaverine, which plays an important role in pH homeostasis by neutralizing the acidic by-products of carbohydrate fermentation. In order to explore the mechanism that *cadBA* contributes to acid tolerance of *E. tarda*, we examined the effect of *cadBA* on activity of lysine decarboxylase. The LDC assay was performed and the result of qualitative measurement shows that TX01 exhibited observable purple after incubation in static culture for 10 h, which indicates an LDC positive phenotype (Figure 3A). However, three mutants exhibited faint yellow or light color, which indicates an LDC negative phenotype. After culturing for 20 h, TX01 exhibited a stronger purple and TX01Δ*cadA* and TX01Δ*cadB* displayed observable purple, but TX01Δ*cadBA* remained faint yellow. When culturing time reaches 30 h, all four strains displayed different degrees of purple. Complementary strains TX01Δ*cadA*C and TX01Δ*cadB*C could partially recover the positive phenotype of lysine decarboxylase (Figure 3A). The quantitative assays revealed that the lysine decarboxylase activity in the mutant TX01Δ*cadA*, TX01Δ*cadB*, and TX01Δ*cadBA* was 10.1, 26.2, 3.4 U, respectively, which all are significantly lower than that in TX01 (34.6 U). Among the three mutants, the lysine decarboxylase activity of TX01Δ*cadBA* was significantly lower than that of TX01Δ*cadA*, and the latter was significantly lower than that of TX01Δ*cadB* (Figure 3B)*.* The lysine decarboxylase activity of complementary strain TX01Δ*cadB*C was similar to that of TX01, while that of complementary strain TX01Δ*cadA*C was dramatically higher than that of TX01Δ*cadA* but a little lower than that of TX01 (Figure 3B).

### *cadBA* mutations enhance bacterial biofilm formation, notably under acid conditions

**Figure 4:**
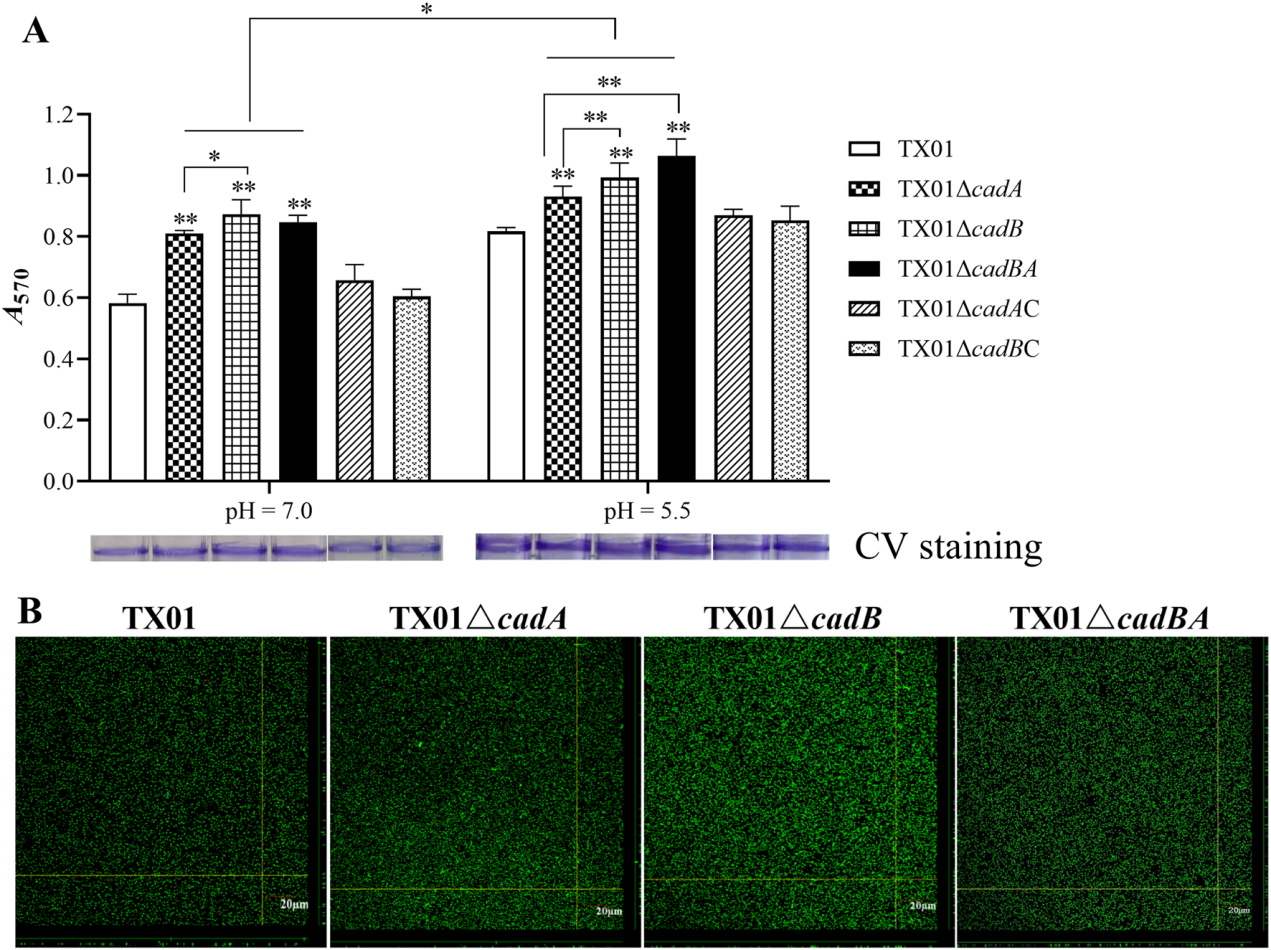
**Effects of *cadBA* mutations on biofilm formation.A** Biofilm-forming capacity of *E. tarda* in normal and acid conditions. TX01, TX01Δ*cadA*, TX01Δ*cadB*, TX01Δ*cadBA*, TX01Δ*cadA*C, and TX01Δ*cadB*C were inoculated into LB broth at pH = 7.0 and 5.5, then incubated in polystyrene plates for 24 h. Biofilm formations were determined by measuring the *A*_570_ of the final eluates of crystal violet staining. Data are presented as the means ± SEM (*N* = 3). N, the number of times the experiment was performed. **, *P* < 0.01; *, *P* < 0.05. **B** The viability of biofilm growth of *E.tarda* was determined by confocal laser scanning microscopy (CLSM). Cells in the biofilms were stained with a BacLight LIVE/DEAD kit to reveal viable (green fluorescence) and non-viable (red fluorescence) bacteria.

Bacterial growth in biofilm mode enhances microbial survival and safeguards them from environmental stresses, including acid stress. We want to know whether the defect in acid tolerance caused by the deletion of *cadBA* affects bacterial biofilm growth, so the biofilm formations of TX01, TX01Δ*cadA*, TX01Δ*cadB*, TX01Δ*cadBA*, TX01Δ*cadA*C, and TX01Δ*cadB*C were determined by CV staining. As shown in Figure 4A, the biofilm forming capabilities of TX01Δ*cadA*, TX01Δ*cadB*, and TX01Δ*cadBA* were significantly stronger than that of TX01 when bacteria were cultured in normal LB medium (pH = 7.0) for 24 h. Consistently, images of the biofilms of four strains obtained by confocal laser scanning microscopy (CLSM) also showed that thicknesses and densities of the biofilm of three mutants were stronger than the wild type, and thickness and density of TX01Δ*cadB* was the strongest amongst three mutants (Figure 4B). When bacteria were cultured in acid LB medium (pH = 5.5), the biofilm growths of all the strains exhibited a significant increase, especially TX01Δ*cadBA*, compared to those in normal medium (Figure 4A). The complementary strains TX01Δ*cadA*C and TX01Δ*cadB*C restored the capacity of biofilm formation of TX01Δ*cadA*, and TX01Δ*cadB*, respectively*.* These results suggest that *cadBA* negatively participates in biofilm formation, especially under acid conditions.

### *cadBA* mutations impair bacterial motility under acid conditions

**Figure 5:**
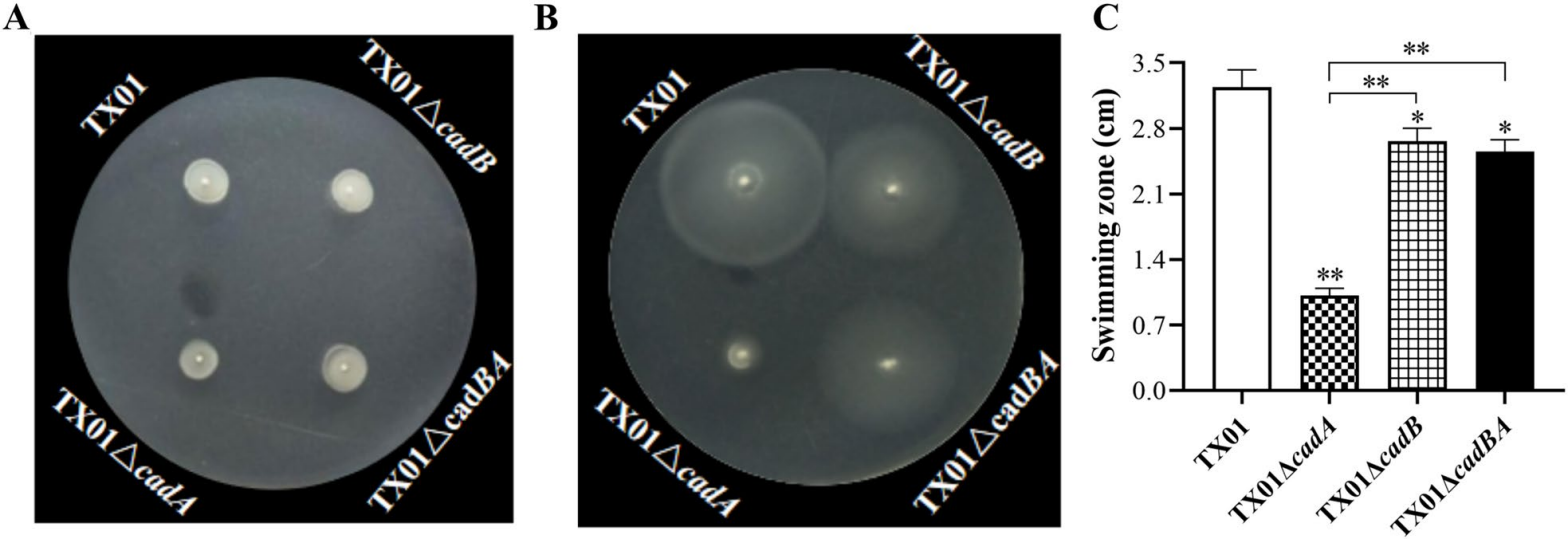
**Effects of *cadBA* mutations on motility of *E. tarda*.** TX01, TX01Δ*cadA*, TX01Δ*cadB*, and TX01Δ*cadBA* were cultured in LB medium to an OD_600_ of 0.5, then aliquots of cell suspensions (1 μL) were inoculated into the center of swimming plates including 0.3% (W/V) agar with pH = 7.0 (**A**) or pH = 5.5 (**B**) at 28 °C for 18 h. **C**, The diameter of the swimming zone from the swimming plate at pH = 5.5. Data are presented as the means ± SEM (*N* = 3). N, the number of times the experiment was performed. **, *P* < 0.01; *, *P* < 0.05.

To investigate whether deletion of *cadBA* has any effect on bacterial motility, six strains were inoculated into swimming plates and bacterial motilities were observed for 18 h. The swimming assay shows that on the neutral LB plate, TX01 and three mutants exhibited similar and little swimming zones (Figure 5A). However, in the acid swimming plate except TX01Δ*cadA*, motility remained weak, other three strains’ moveability were obviously enhanced (Figure 5B), but the swimming zones of TX01Δ*cadB* and TX01Δ*cadBA* were significantly shorter than that of TX01 (Figure 5C). The swimming zone of complementary strain TX01Δ*cadB*C was comparative to that of TX01, while the swimming zone of complementary strain TX01Δ*cadA*C was shorter than that of TX01 (Additional file 3), which indicates TX01Δ*cadA*C could have partially recovered swimming ability in the acid swimming plate. These results suggest that *cadBA* operon mutations impair *E. tarda*'s motility under acid conditions.

### *cadBA* deletions reduce the ability of adhesion and invasion to HeLa cells

**Figure 6:**
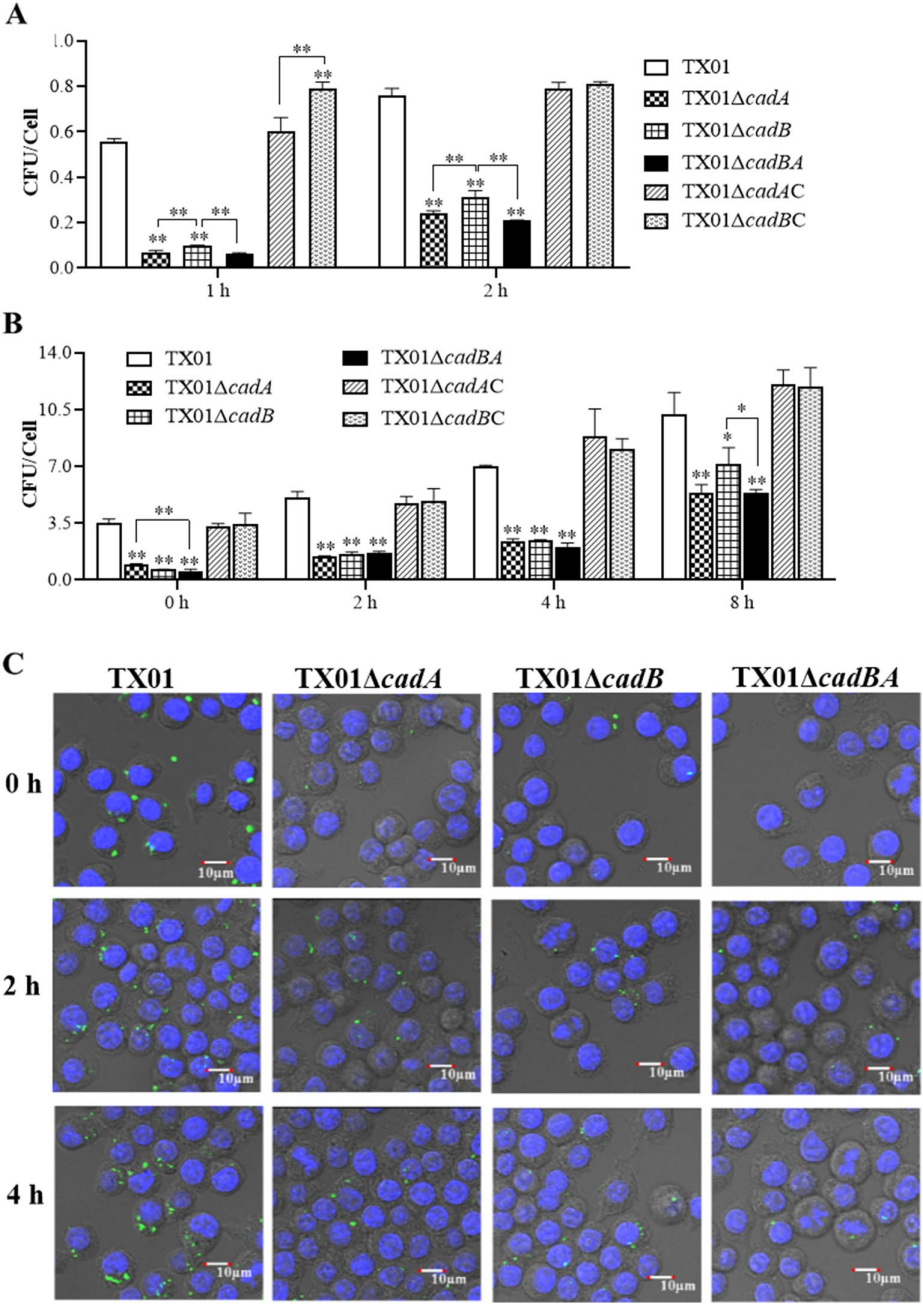
**Effects of *cadBA* mutations on cellular infection and replication.A** Invasion of HeLa cells by *E. tarda*. HeLa cells were infected with the same dose of *E. tarda* TX01, TX01Δ*cadA*, TX01Δ*cadB*, TX01Δ*cadBA*, TX01Δ*cadA*C and TX01Δ*cadB*C strains for 1 and 2 h, and washed with PBS. Then, HeLa cells were lysed and the CFU were counted. **B** Replication of *E. tarda* in macrophages. The murine macrophage cell line RAW264.7 was infected with *E. tarda* and mutants above mentioned for 2 h, followed by treatment with gentamicin for 2 h to kill extracellular bacteria. After being washed with PBS, the cells were incubated for the time intervals indicated. Then, the cells were lysed and the CFU were counted. Data are the means of three independent experiments and presented as means ± SEM (*N* = 3). N, the number of times the experiment was performed. **, *P* < 0.01; *, *P* < 0.05. **C,** TX01, TX01Δ*cadA*, TX01Δ*cadB* and TX01Δ*cadBA* containing pGFPuv plasmid were used to infect RAW264.7 cells for 0, 2 and 4 h. DNA was stained blue by DAPI, the cells were observed by confocal microscopy.

The above results show that the deficiency of *cadBA* increased *E. tarda*’s biofilm formation but decreased motility in an acid environment, and reduced adversity resistance. These physiological phenotypes are all related to bacterial pathogenicity, so we want to know whether the deletion of *cadBA* could affect the virulence of *E. tarda*. To evaluate the participation of *cadBA* operon in the virulence of *E. tarda*, HeLa cells were infected with the same dose of TX01Δ*cadA*, TX01Δ*cadB*, TX01Δ*cadBA*, TX01Δ*cadA*C, and TX01Δ*cadB*C strains for 1 and 2 h, then were washed three times and lysed. The bacteria adhering to the surface of cells and invading into the interior were examined. The results of plate counting indicate that the amounts of TX01Δ*cadA*, TX01Δ*cadB*, and TX01Δ*cadBA* from HeLa cells were remarkably less than that of TX01 at 1 and 2 h post-infection, and the amounts of TX01Δ*cadA* and TX01Δ*cadBA* from HeLa cells were also significantly less than that of TX01Δ*cadB* at both time points (Figure 6A). The amounts of complementary strain TX01Δ*cadA*C from HeLa cells were comparative to those of TX01. However, the amount of complementary strain TX01Δ*cadB*C from HeLa cells was more than that of TX01 at 1 h post-infection, but the amounts of both were comparative at 2 h post-infection. These observations suggest that the mutations of *cadBA* impair the adhesion and invasion of *E. tarda* to non-phagocytes.

### *cadBA* is required for bacterial survival and replication in macrophages

*E. tarda* prefers an intracellular lifestyle in host cells to escape the innate immunity and to multiply. Next, we examined the role of *cadBA* in phagocyte survival and replication of *E. tarda*. The same dose of TX01, TX01Δ*cadA*, TX01Δ*cadB*, TX01Δ*cadBA*, TX01Δ*cadA*C, and TX01Δ*cadB*C were used to infect murine macrophage cell line RAW264.7. After eliminating extracellular bacteria, RAW264.7 cells were cultured for different hours and were lysed to determine the intracellular bacteria. From the results of plate counting, we found that the amounts of TX01Δ*cadA*, TX01Δ*cadB* and TX01Δ*cadBA* from the intracellular macrophage cells were remarkably less than those of TX01 at 0, 2, 4, and 8 h post-infection, and the amount of TX01Δ*cadBA* was basically the least amongst the three mutants (Figure 6B). The amounts of complementary strains TX01Δ*cadA*C and TX01Δ*cadB*C from the intracellular macrophage cells were similar to those of TX01.

To observe the living bacteria in macrophages, *E. tarda* containing pGFPuv plasmid was used to infect RAW264.7 cells. By means of confocal microscopy, it was clearly observed that the fluorescence intensities from TX01Δ*cadA*-, TX01Δ*cadB*-, and TX01Δ*cadBA*-infected macrophages were weaker than those from TX01-infected cells at 0, 2 and 4 h post-infection (Figure 6C). These results suggest that the mutations of *cadBA* impair the survival and replication of *E. tarda* in host phagocytes.

### *cadBA* is a requisite for bacterial dissemination in host tissues

**Figure 7:**
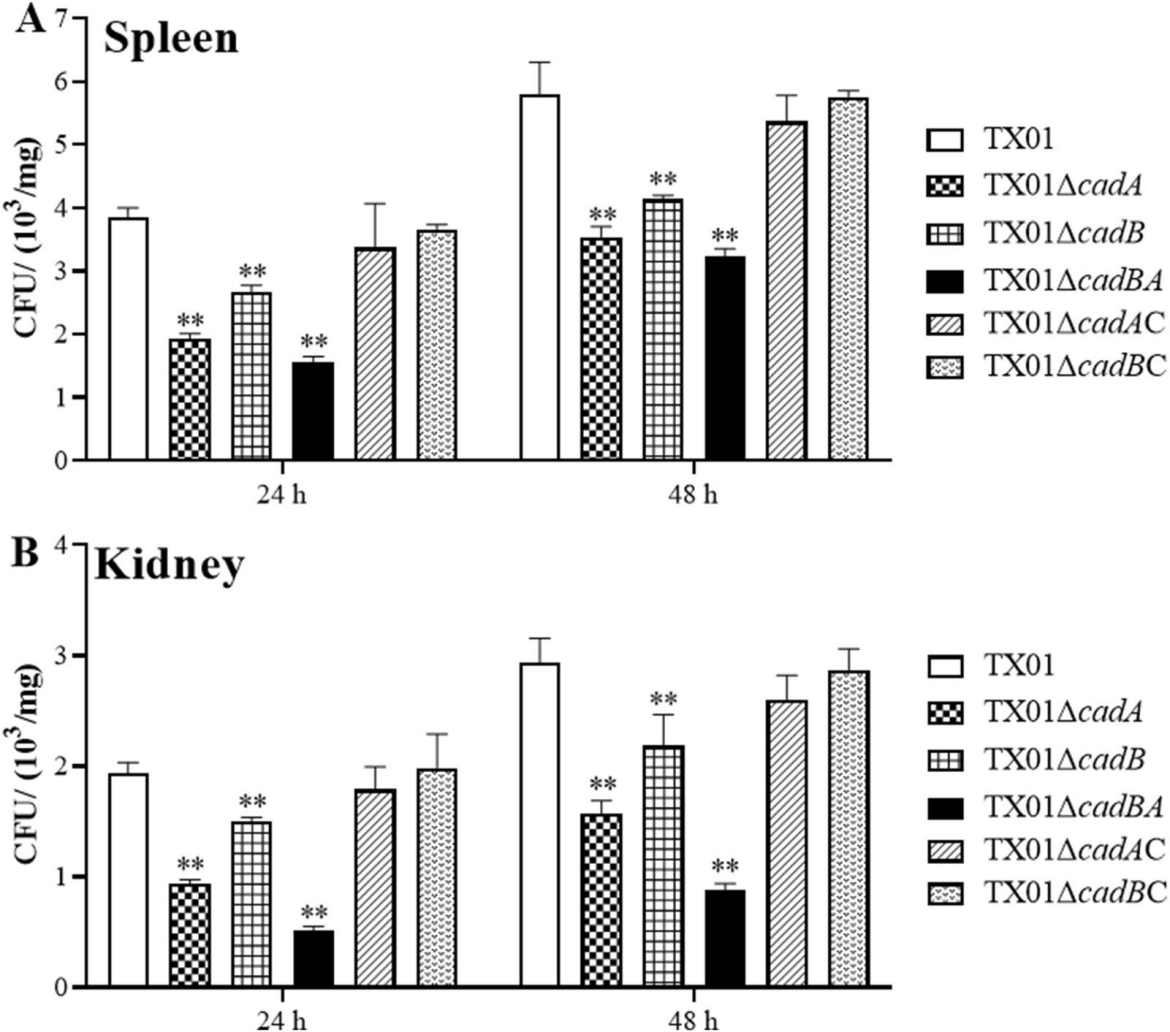
**Bacterial dissemination in the fish tissues.** Tilapia were infected with the same dose of TX01, TX01Δ*cadA*, TX01Δ*cadB*, TX01Δ*cadBA*, TX01Δ*cadA*C, and TX01Δ*cadB*C. The recoveries of bacteria in spleen (**A**) and kidney (**B**) were determined by plate counting at 24 and 48 h post-infection. Data are presented as means ± SEM (*N* = 3). N, the number of times the experiment was performed. **, *P* < 0.01.

An in vitro experiment has confirmed the participation of *cadBA* operon in *E. tarda*’s invasion of host cells. Next, in vivo experiments were performed to determine the contribution of *cadBA* operon to virulence of *E. tarda.* To do this, the same doses of strains (TX01, TX01Δ*cadA*, TX01Δ*cadB*, TX01Δ*cadBA*, TX01Δ*cadA*C, and TX01Δ*cadB*C) were used to infect tilapia. Viable bacteria from spleen and kidney were determined by plate counting at 24 and 48 h post-infection. The results show that bacterial amounts in both tissues from TX01Δ*cadA*-, TX01Δ*cadB*- and TX01Δ*cadBA*-infected tilapias were obviously less than those from TX01-infected tilapias at both infection time points. And the amounts of two complementary strains TX01Δ*cadA*C and TX01Δ*cadB*C from infected tilapias were basically equal to those of TX01 (Figure 7). These results imply that the *cadBA* operon is involved in the dissemination of *E. tarda* in the fish tissue.

### *cadBA* expression is up-regulated upon acid stress

**Figure 8:**
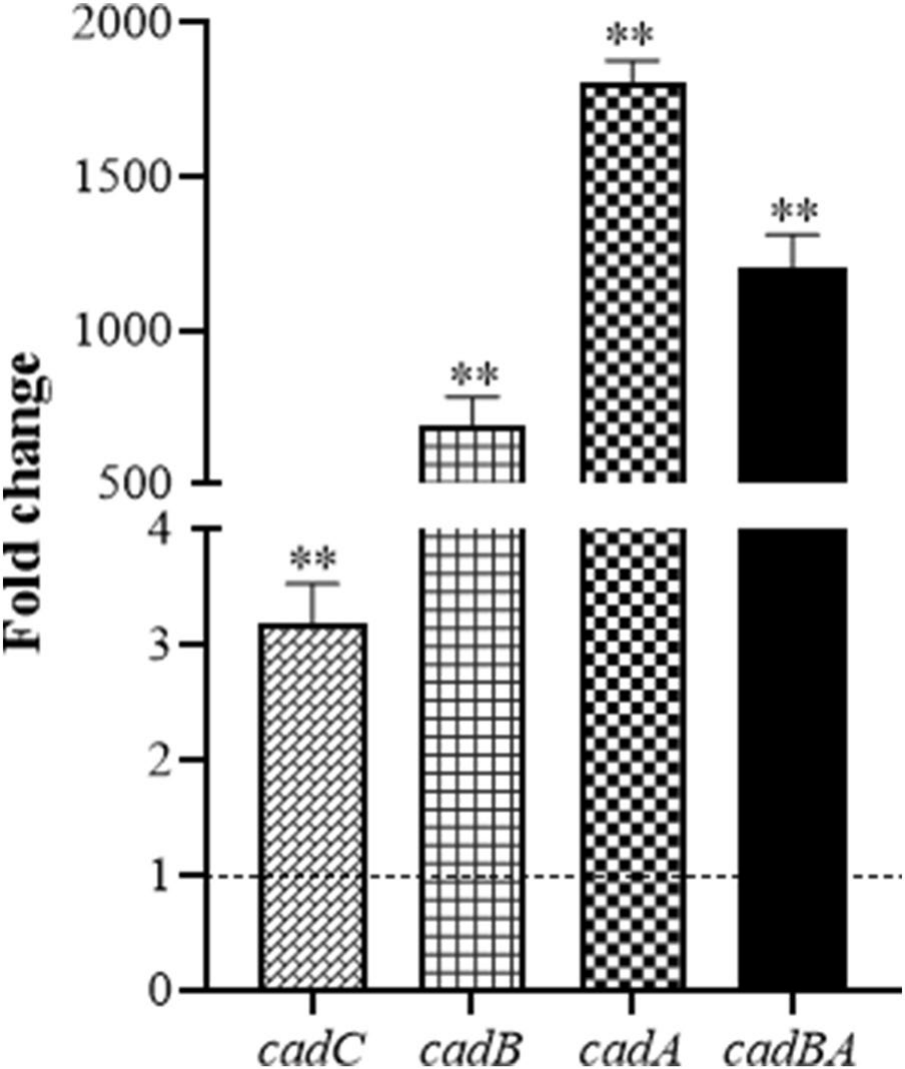
**The expression of acid resistance genes from TX01 under acidic conditions.** The exponential phase of overnight cultures of TX01 were grown in normal LB medium at pH 7.0 and acidic medium at pH 5.5 for 1 h. The relative expression of *cadB*, *cadA*, *cadBA*, and *cadC* were determined by RT-qPCR. The fold difference derived from the values under acidic conditions compared with the values under normal conditions. Data are presented as the means ± SEM (*N* = 3). N, the number of times the experiment was performed. **, *P* < 0.01; *, *P* < 0.05.

Since *cadBA* was closely related to acid tolerance, we wanted to survey the expression of *cadBA* under acid pressure. *E. tarda* in exponential phase were subjected to an acidic medium (pH 5.5) for 1 h, then RNA was extracted and cDNA was obtained. RT-qPCR shows that the expression of *cadB*, *cadA*, and *cadBA* was up-regulated 685.5-, 1808.7-, and 1204.1-folds respectively, upon acid stimulation, which was significantly higher than that of the control (Figure 8). The immediate upstream of *cadBA* is *cadC*, so we also surveyed its expression and found that *cadC* expression was significantly enhanced (3.2-folds) by acid stimulation (Figure 8). These results indicate that the *cadBA* operon is closely involved in acid tolerance of *E. tarda*.

### *cadBA* expression is regulated by CadC

**Figure 9:**
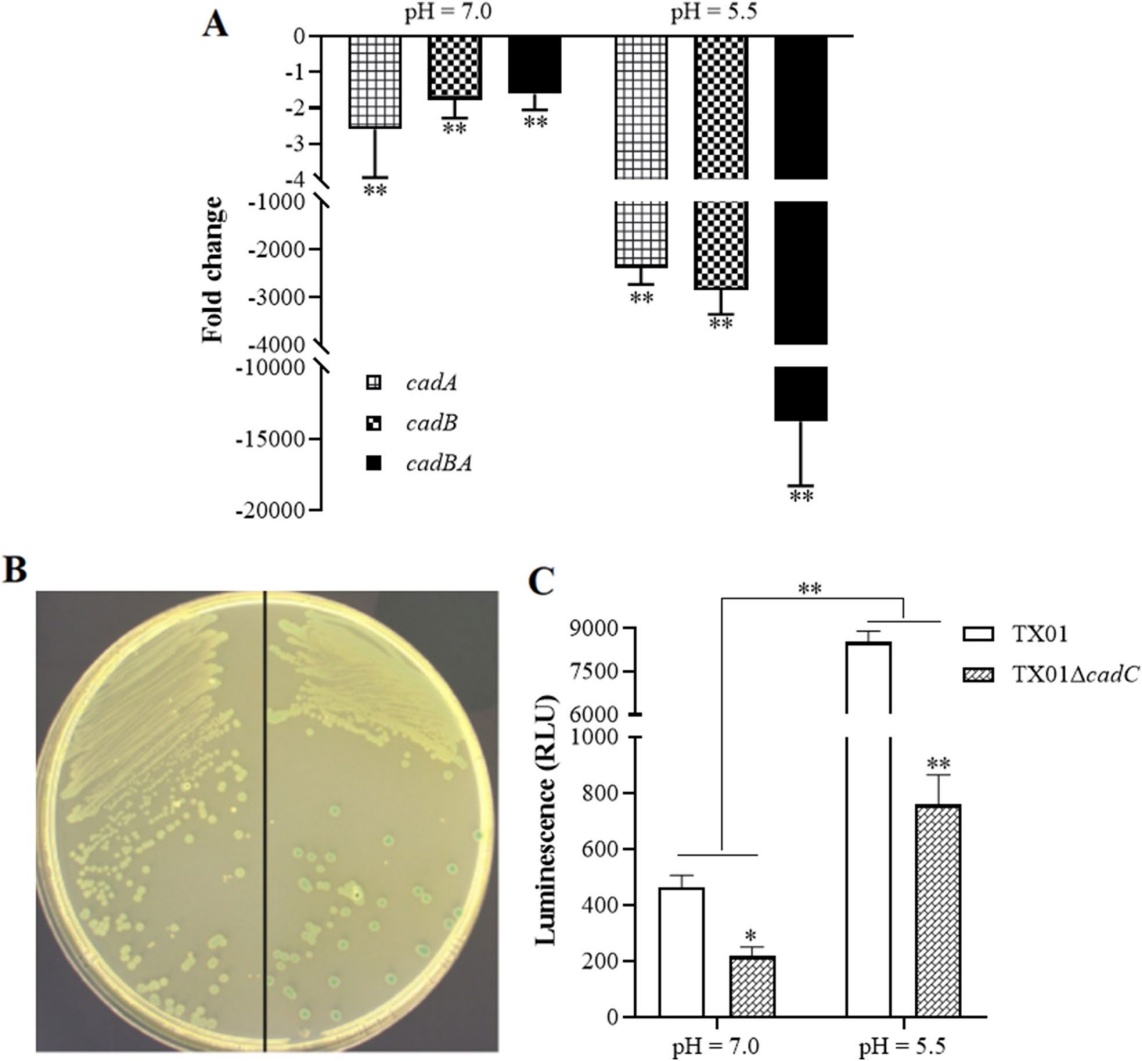
**Transcriptional regulation of *cadBA* by CadC in vitro or in vivo.A** Expression profiles of *cadBA* operon in normal and acidic conditions. *E*. *tarda* TX01 and TX01Δ*cadC* in logarithmic growth phase was culture in normal LB (pH = 7.0) or in acidic LB (pH = 5.5) for 1 h, then the expression of *cadB*, *cadA*, and *cadBA* were examined by RT-qPCR. For convenience of comparison, the expression level of *cadBA* operon in wild type TX01 set as 1. **B** The promoter activity of *cadBA* operon was regulated by CadC in vitro. DH5α/pSC418/pJR21 and DH5α/pSC418/pJR21C were streaked and cultured on an X-gal plate at pH 5.5 in the presence of 5 mM L-lysin. The depth of blue indicates the strength of promoter activity. **C** The promoter activity of *cadBA* operon was regulated by CadC in vivo. The wild-type TX01 and TX01Δ*cadC* mutant carrying the reporter plasmid pJR21-418-lx were cultured to the exponential phase. Then strains were transferred acid LB media (pH = 5.5) supplemented with 5 mM l-lysine and grown for 1 h. The transcriptional regulation of promoter of *cadBA* was assessed by measuring luciferase activity. Data are the means of three independent experiments and presented as means ± SEM (*N* = 3). N, the number of times the experiment was performed. **, *P* < 0.01; *, *P* < 0.05.

Since *cadC* is located upstream of the *cadBA* operon and defined as a transcriptional regulator, and the expression of *cadC* was induced by acid stimulation, we speculated that the expression of *cadBA* is regulated by CadC. In another study, we obtained *a cadC* mutant (named TX01Δ*cadC*). We first investigated the expression of *cadBA* in wild strain TX01 and TX01Δ*cadC* under normal conditions and acidic conditions. RT-qPCR shows that under normal conditions, the expression of *cadA*, *cadB*, and *cadBA* in TX01Δ*cadC* was significantly lower than that in TX01, the values were 2.6-, 1.8-, and 1.6-fold, respectively. Under acidic conditions, the expression of *cadA*, *cadB*, and *cadBA* in TX01Δ*cadC* was down regulated by 2382.0-, 2848.0-, and 13,777.6-folds respectively, compared to TX01 (Figure 9A). These results support the conclusion that CadC is an activator of *cadBA* transcription, and its regulator role is mainly displayed under acidic conditions.

To detect the regulatory mechanism of CadC on *cadBA*, the speculative promoter of *cadBA*, P418, was cloned to a promoter probe plasmid pSC11 resulting in DH5α/pSC418. Meanwhile, the expression plasmid of *cadC* was constructed and named pJR21C. DH5α/pSC418 cultured on X-gal plate at pH 7.0 and 5.5 in the presence of 5 mM l-lysine shows a weak blue phenotype, indicating that the P418 promoter has a weak promoter activity. When pJR21C was transformed into DH5α/pSC418, the blue color of the recombinant strain (DH5α/pSC418/pJR21C) on the X-gal plate at pH 7.0 remained unchanged in the presence of 5 mM l-lysine, compared to that of the control (DH5α/pSC418/pJR21) (data not shown). However, the blue color of DH5α/pSC418/pJR21C on X-gal plate at pH 5.5 in the presence of 5 mM l-lysine was obviously enhanced, compared to that of the control (Figure 9B). These results suggest that CadC positively regulates the promoter activity of *cadBA* under acidic conditions.

To further analyze the regulation of CadC on *cadBA* in vivo, promoter reporter plasmid pJR21-418-lx was introduced into TX01 and TX01△*cadC*, and the luminescence induced by promoter P418 was detected. The results show the relative light units of (RLU) of TX01 were 463.5 U and 8513.58 U under normal conditions and acidic induced conditions, respectively, which both are significantly higher than that in TX01△*cadC* (213.7 U and 757.95 U, respectively), especially under acidic induction conditions (Figure 9C). These results indicate that CadC positively regulates the transcription of *cadBA* in *E. tarda*, especially under acidic conditions.

## Discussion

Pathogenic and commensal bacteria often encounter a lot of strong and mild acidic environments both inside and outside hosts, for example, acid soil and food, the gastrointestinal tract, dental plaque and macrophage phagosome [14]. In general, bacteria are able to maintain a fairly constant internal pH for survival, which is due to evolving acid tolerance response (ATR) and acid resistance (AR) protection mechanisms [17, 36, 42]. One of the important mechanisms involved in acid resistance is the amino acid decarboxylase system that buffers cytosol and the local extracellular environment to ensure enterobacterial survival at low pH [43]. Among them, the lysine-dependent acid resistance (LDAR) system is the mild acid tolerance response system consisting of inducible lysine decarboxylase (CadA) and the lysine/cadaverine antiporter (CadB) [44]. In *E. coli*, the *cadBA* operon contains *cadB* and *cadA* genes, and plays an important role in acid pressure reactions [31]. A similar operon also exists in *Salmonella enteric*, *Vibrio cholera*, and *Vibrio vulnificus* [32, 45, 46]. In *E. tarda*, *cadBA* operon also exists. Sequence analysis shows that CadB is a membrane protein and CadA is an intracellular protein with PLP-binding core domain and the pyridoxal phosphate binding motif. The CadB and CadA of *E. tarda* share high identities with CadB and CadA of *E. coli*, respectively. To date, there are no reports about the LDAR system in *E. tarda*, so we characterized and identified the function of *cadBA* operon in this study.

As a member of Enterobacteriales, *E. tarda* can survive and replicate in an acidic environment, but the underlying mechanisms remain unknown. Although some genes or factors, such as Usp13, TAM, HutZ, TrxH, RpoN, and RpoS have been reported to participate in acid tolerance [37, 47–50], there is no report about the function of traditional or classical acid resistance in *E. tarda*. In this study, our results show that compared to wild type TX01, the growth of three mutants TX01Δ*cadA*, TX01Δ*cadB*, and TX01Δ*cadBA* appeared to be retarded obviously when exposed to an acidic environment (pH = 5.5 to 4.5), especially *cadA* delete mutant TX01Δ*cadA*. Consistently, in *V. parahaemolyticus*, a mutated strain with a disrupted *cadA* gene attenuates acid survival [51]. In *V. vulnificus*, *cadA* mutant accompanying a lack of cadaverine decreases tolerance to low pH [32]. However, in normal medium (neutral pH), the growth rates of TX01 and three mutants are very similar. We observed that the cell densities of TX01Δ*cadA*, TX01Δ*cadB*, and TX01Δ*cadBA* were similar to that of TX01 at the stationary phase, which may be due to the following two reasons: (i) the pH of medium was increased; (ii) another acid resistance system was activated. These results indicate that *cadBA* operon is closely related to acid tolerance of *E. tarda*. In addition, it was reported that microorganisms adapted to mild acid stress may also survive against different types of lethal stress, this kind of multiple adaptive response may be part of a survival strategy [52]. In *E. tarda*, *cadBA* operon also plays a role in resistance against iron deficiency and oxidative stress. These findings indicate that *cadBA* operon is an important factor of stress resistance in *E. tarda*.

It has been demonstrated that cadaverine, decarboxylated from lysine, plays a role in acid survival in *E. coli* [53]. The cadaverine was found to be covalently attached to the cell wall peptidoglycan of bacteria. Inhibition of lysine decarboxylase prevented cadaverine accumulation and incorporation into peptidoglycan, resulting in severe inhibition of cell growth under acidic conditions [54–57]. At acidic pH, CadB functions as a cadaverine/lysine antiporter rather than only cadaverine uptake, which catalyses cadaverine excretion and lysine uptake and generates a membrane potential [21]. CadA is acid-inducible lysine decarboxylase, which produces cadaverine and carbon dioxide and generates a pH gradient through consumption of a cytoplasmic proton, so this proton motive metabolic cycle results in neutralization of acidic conditions [31, 58].

To explore the mechanism that *cadBA* contributes to acid tolerance of *E. tarda*, we applied a simple and convenient bromocresol purple-based colorimetric method for fast qualitative detection of lysine decarboxylase activity. Our results show that all mutants exhibited an LDC negative phenotype at the early stages of culture (before 10 h), which is inconsistent with the results of quantitative analysis. In quantitative analysis, lysine decarboxylase activities of the three mutants followed the trend of TX01Δ*cadBA* < TX01Δ*cadA* < TX01Δ*cadB*. These observations illustrate that *cadBA* operon played a critical role in the decarboxylation of lysine of *E. tarda*, and the LDC activity mainly depended on *cadA*, which was promoted by *cadB*. However, at a later stage of culture (30 h), the LDC activity of three mutants was restored to a certain extent, suggesting another minor decarboxylation reaction may be activated when *cadBA* operon was inactivated. Unlike *E. coli* and *V*. *cholerae* [59, 60], the *E. tarda* genome possesses three genes (ETAE_0284, ETAE_0757, and ETAE_3006) encoding lysine decarboxylase and three genes (ETAE_0283, ETAE_0756, and ETAE_1884) encoding lysine/cadaverine antiporter. RT-qPCR results show that the genes ETAE_3006 and ETAE_1884 were also up-regulated under acidic conditions. However, they do not form the operon, the expression of gene level was lower than that of the *cadBA* operon (Additional file 4). We speculated that ETAE_3006 and ETAE_1884 could remedy the deficiency of CadBA to a certain extent at a later stage.

Acid resistance is closely related to biofilm formation. Biofilms are a kind of microbial aggregates embedded in a self-generated matrix of extracellular polymeric substances, by which the microbes can adhere to each other or to the surface of material [61–63]. It has been reported that biofilm formation strengthened bacterial tolerance to acid stress [64]. For example, biofilm formation significantly increased acid tolerance in clinical streptococcal strains, such as *Streptococcus gordonii*, and *S. oralis* [65]. Complex biofilms were formed when *Pseudomonas aeruginosa* was exposed to external acid stress [66]. In this study, we examined the relationship between acid resistance system CadBA and biofilm formation. The results show that whether wild strain TX01 or the three mutants TX01Δ*cadA*, TX01Δ*cadB*, and TX01Δ*cadBA*, their abilities to form biofilms was significantly enhanced when bacteria were exposed to acid stress, which indicates biofilm is needed for bacterial resistance against acid stress. However, the partial and total inactivation of *cadBA* operon significantly increased *E. tarda*’s capability of biofilm growth under normal conditions (pH = 7.0) and under acidic conditions (pH = 5.5). Moreover, the biofilm increase by the inactivation of *cadBA* operon under acidic conditions was higher than that under normal conditions. These results illustrate that *cadBA* operon negatively participated in the biofilm formation. Different from *cadBA*, *hutZ* mutation and *usp13* mutation in *E. tarda* decreased both bacterial resistance against acid stress and biofilm formation [37, 47]. YefM-YoeB operon, a type II TA system, is not related to acid tolerance, but its mutation enhanced biofilm formation of *E. tarda* [34]. In *Streptococcus Mutans*, urethane dimethacrylate promotes biofilm formation but reduces acid tolerance [67], which is the opposite to the effect of *cadBA* in *E. tarda*. This study revealed for the first time, CadBA’s involvement in bacterial biofilm formation in an acid resistance system. It was obvious that the relationship between biofilm and acid resistance is complex, and the mechanism is still poorly understood.

The above findings clearly demonstrate that the *cadBA* operon was involved in adversity resistance and biofilm formation, which are related to bacterial pathogenicity. It has been reported that *cadBA* operon participates in virulence. In *E. coli*, CadA has been proposed to negatively regulate virulence in several enteric pathogens [68, 69]. When *cadA* was introduced into *Shigella flexneri*, bacterial virulence became attenuated, and enterotoxin activity was greatly inhibited [70]. In contrast, isogenic deletion of *cadA* in *S. pneumoniae* TIGR4 led to attenuation of colonization, pneumococcal pneumonia and sepsis in murine models [71]. The toxicity of Δ*cadB* of *Aeromonas veronii* was 9.5 times less than that of TH0426 in EPC cells [72]. In this study, our results show that the partial or total inactivation of *cadBA* operon significantly weakened the ability of *E. tarda* to adhere and invade host non-phagocytes. Complementary strain TX01Δ*cadA*C recovered the lost virulence of TX01Δ*cadA*. Curiously, complementary strain TX01Δ*cadB*C exhibited stronger infection ability than the wild type strain at 1 h post-infection, but was back to the TX01level at 2 h post-infection. We also noted that TX01Δ*cadA*C partially rescued the lost lysine decarboxylase activity of TX01Δ*cadA*. The reason for the difference exhibited by TX01Δ*cadA*C is worth exploring in the future.

Similarly, the capability of *E. tarda* to survive and replicate in host macrophage cells was significantly declined when the *cadBA* operon was partially or completely deleted. An in vivo experiment also supported the conclusion that the *cadBA* operon contributed to the virulence of *E. tarda*. These findings illustrate that the CadBA acid resistance system plays an important role in virulence of *E. tarda*. Acid stress, as well as iron deficiency and oxidative stress, are important environmental pressures in the host. CadBA promoted the resistance of *E. tarda* to these adversities, and enhanced bacterial motility, thereby contributing to *E. tarda*’s invasion to host cells and the host.

Since *cadBA* operon is important for adversity resistance and pathogenicity, its expression is precisely regulated. It is reported that the *cadBA* operon is induced at acid conditions coupled with lysine [60, 73]. In this study, we found that the expression of *cadBA* operon was remarkably induced by acid stress. For *cadA* and *cadBA*, their expressions were induced more than 1000 times. This result supports the conclusion that the LDAR system CadBA in *E. tarda* is important for bacterial acid adaption. It is worth noting that the transcriptional expressions of *cadB*, *cadA*, and *cadBA* were different, although *cadB* and *cadA* are bicistron. Among the abundance of three transcriptions under acid stress, *cadA* was the highest, *cadBA* the second, and *cadB* the lowest. Similar results were observed in *Vibrio parahaemolyticus* [51]*.* We speculated that there is another promoter before the ORF of *cadA*. In addition, *cadC*, the upstream gene of *cadBA*, was also induced by acid stress, although its expression was only three folds. Like most *cadBA* operons in Enterobacteriaceae, CadC was the regulator of *cadBA* operon. To affirm the regulator role of CadC in *E. tarda*, the expression of *cadBA* operon was examined in the *cadC* mutant. RT-qPCR shows that when *cadC* was deleted, the expression of the *cadBA* operon was down-regulated, especially under acid conditions, the expression of *cadBA* operon was down-regulated by 2382- to 13,778-folds, which also indicates the involvement of *cadBA* operon in acid tolerance. The assay of detection of promoter activity in vitro and in vivo suggests that CadC regulated the promoter activity of *cadBA* operon. These results illustrate that the expression of *cadBA* operon was strictly regulated by CadC, especially under acid conditions.

In conclusion, for the first time we identified and characterized the *cadBA* operon in *E. tarda*, a significant zoonotic pathogen. Our results show that the *cadBA* operon plays an important role in coping with adverse circumstances, especially acid stress. It is the first time that the *cadBA* operon negatively participated in biofilm formation, especially under acid conditions. Our results also demonstrate that *cadBA* functions as a new virulence factor that is essential to bacterial infection at the cellular and tissue levels. The expression of *cadBA* operon was induced upon acid stress by the regulator CadC. This study provides new insight into the acid resistance mechanism of *E. tarda*.

## Supplementary Information

**Additional file 1.Sequence alignment of CadA (A) and CadB (B) homologues.** Gaps used to maximize the alignment are indicated by dots. The complete conserved amino acid residues are shown in dark blue. The amino acid residues with conservative degree higher than 75% are shown in pink. The black line indicates the N-terminal domain. The green and orange lines indicate the PLP-binding core domain and C-terminal domain, respectively. Red triangles indicate PLP binding sites. Conserve PLP binding motif is yellow boxed. The blue line indicates transmembrane (TM) domains. The GenBank accession numbers of CadA homologues are as follows: *Edwardsiella tarda*, ACY83602.1; *Escherichia coli* str. K-12 substr, NP_418555.1; *Salamae*, VEA01344.1; *Aeromonas veronii*, QHC07268.1; *Vibrio cholerae*, WP_188372281.1; *Klebsiella pneumoniae*, AZH95435.1; *Shigella boydii*, PHU85906.1. The GenBank accession numbers of CadB homologues are as follows: *E. tarda*, ACY83601.1; *Salmonella enterica*, WP_000100010.1; *Klebsiella pneumoniae*, WP_110924908.1; *Escherichia coli*, WP_175119720.1; *Shigella flexneri*, NP_709997.1; *Vibrio cholerae*, WP_189017881.1; *Aeromonas veronii*, WP_118881724.1.

**Additional file 2.Validation of TX01 mutants by PCR amplification.** Lanes 1 and 7 were the 661-bp (deletion of *cadA* allele) fragments amplified from TX01Δ*cadA* and TX01Δ*cadBA* genomic DNA with primers CadAKOF3/CadAKOR3, respectively. Lane 4 was 1684-bp *cadA* fragment*.* Lanes 5 and 8 were the 265 bp (deletion of *cadB* allele) fragments amplified from TX01Δ*cadB* and TX01Δ*cadBA* genomic DNA with primers CadBKOF3/CadBKOR3, respectively. Lane 2 was 901-bp *cadB* fragment. Lanes 3, 6 and 9 were 16S rRNA gene fragments amplified from three mutants with universal primers 27F/1492R. M is DS 2000 marker.

**Additional file 3.The swimming ability of complementary strains TX01Δ*cadA*C and TX01Δ*cadB*C in the acid swimming plate.** TX01, TX01Δ*cadA*C, and TX01Δ*cadB*C were cultured in LB medium to an OD_600_ of 0.5, then aliquots of cell suspensions (1 μL) were inoculated into the center of swimming plates including 0.3% (W/V) agar with pH = 5.5 at 30 °C for 18 h (**A**). The diameter of the swimming zone from the swimming plate at pH = 5.5 (**B**). The data are presented as the means ± SEM (*N* = 3). N, the number of times the experiment was performed. **, *P* < 0.01.

**Additional file 4.The expression of acid resistance genes in *Edwardsiella tarda* under acidic conditions.** The exponential phase of overnight cultures of TX01 were grown in normal LB medium at pH 7.0 and acidic medium at pH 5.5 for 1 h. The relative expression of ETAE_3006 and ETAE_1884 were determined by RT-qPCR. The fold difference derived from the values under acidic conditions compared with the values under normal conditions. Data are presented as the means ± SEM (*N* = 3). N, the number of times the experiment was performed. **, *P* < 0.01.

## Data Availability

All data generated or analyzed during this study are included in this published article.

## Abbreviations

*E. tarda*: *Edwardsiella tarda*
*E. anguillarum*: *Edwardsiella* *anguillarum*
*E. **ictaluri*: *Edwardsiella* *ictaluri*
*E. hoshinae*: *Edwardsiella* *hoshinae*
*E. piscicida*: *Edwardsiella* *piscicida*
*E. coli*: *Escherichia coli*
*V. cholera*: *Vibrio cholera*
*S*. *typhimurium*: *Salmonella typhimurium*
*V. vulnificus*: *Vibrio vulnificus*
AR: acid-resistance
ATR: acid tolerance response
PLP: phosphopyridoxal
GDAR: glutamic acid-dependent acid resistance
ADAR: arginine-dependent acid resistance
LDAR: lysine-dependent acid resistance
ODAR: ornithine-dependent acid resistance
LB: Luria–Bertani broth
PCR: Polymerase Chain Reaction
RT-PCR: reverse transcription-PCR
RT-qPCR: quantitative real-time reverse transcription-PCR
ORF: open reading frames
CFU: colony forming unit
Dp: 2,2ʹ-Dipyridyl
LDC: lysine decarboxylase
OD: optical density
*A*: absorption
CV: crystal violet
CLSM: confocal laser scanning microscopy
PBS: phosphate-buffered saline
DMEM: Dulbecco’s Modified Eagle’s medium
MOI: multiplicity of infection
DAPI: 4ʹ, 6-Diamidino-2-phenylindole
X-gal: 5-Bromo-4-chloro-3-indolyl-beta-d-galactopyranoside
U: unite
TM: transmembrane domains
i.m.: intramuscular
MS222: tricaine methanesulfonate
