# Supplementary figures and images for: Acid resistance system CadBA is implicated in acid tolerance and biofilm formation and is identified as a new virulence factor of Edwardsiella tarda
Source: Vet Res. 2021 Sep 14;52:117. doi: 10.1186/s13567-021-00987-x (PMC8438976; doi:10.1186/s13567-021-00987-x)

**
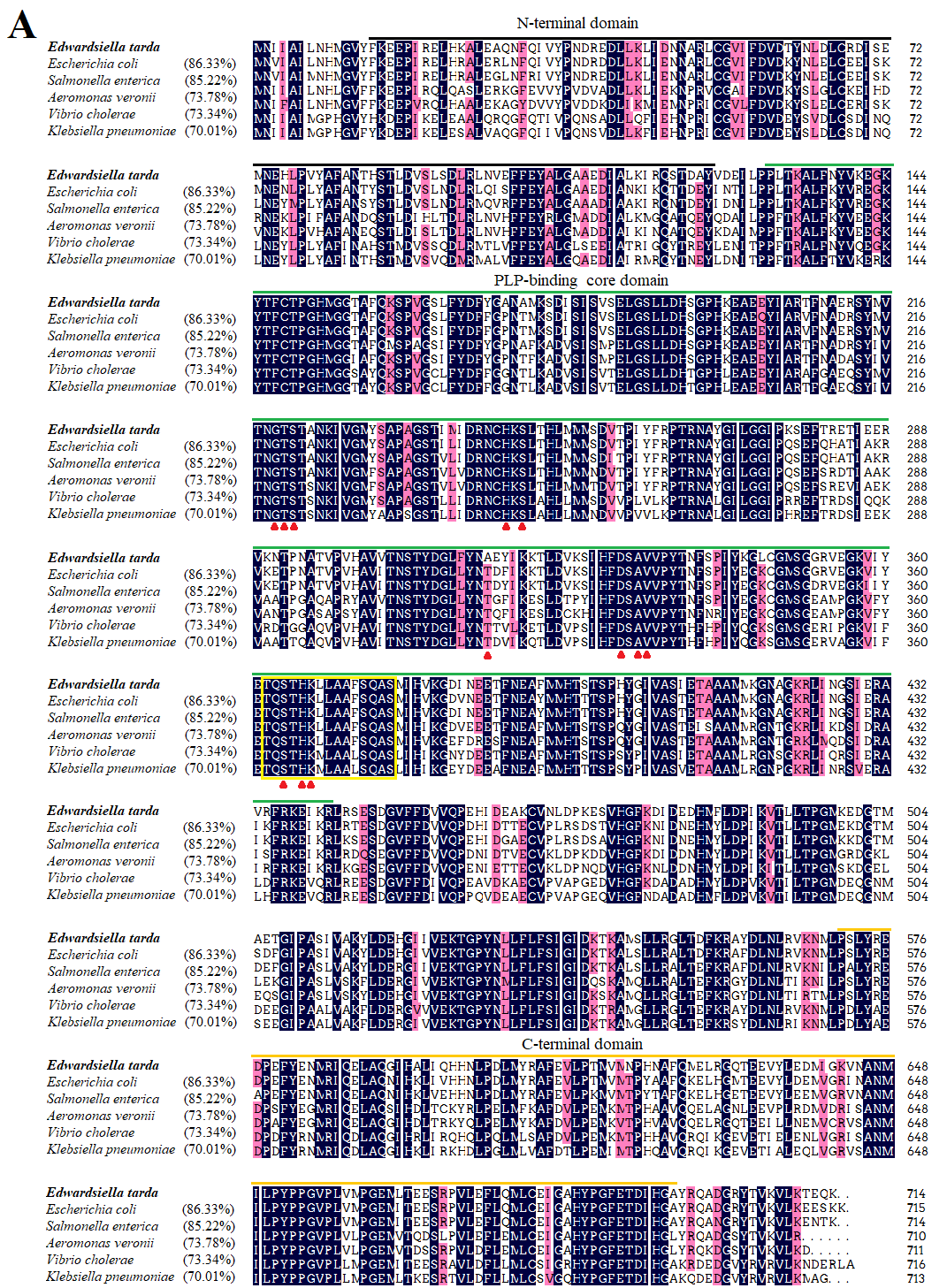
**

**
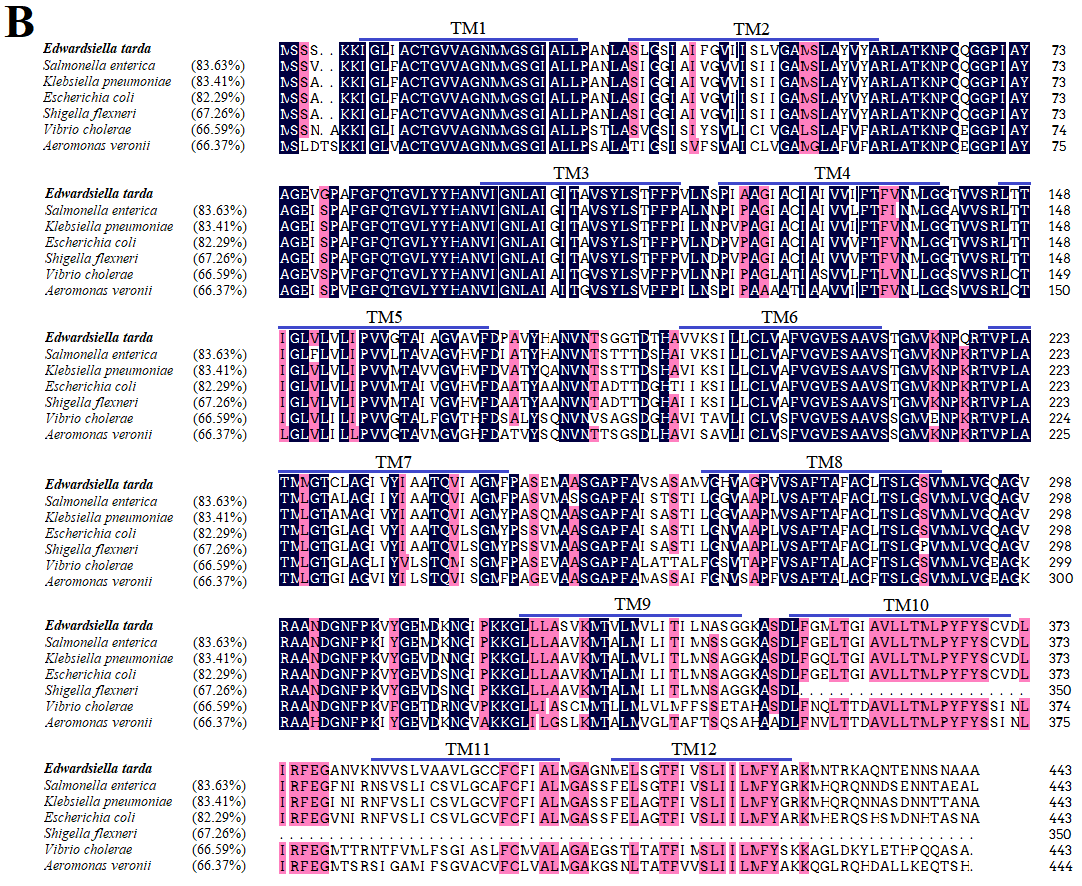
**

Supplement: Supplementary file 1 — Additional file 1.Sequence alignment of CadA (A) and CadB (B) homologues. Gaps used to maximize the alignment are indicated by dots. The complete conserved amino acid residues are shown in dark blue. The amino acid residues with conservative degree higher than 75% are shown in pink. The black line indicates the N-terminal domain. The green and orange lines indicate the PLP-binding core domain and C-terminal domain, respectively. Red triangles indicate PLP binding sites. Conserve PLP binding motif is yellow boxed. The blue line indicates transmembrane (TM) domains. The GenBank accession numbers of CadA homologues are as follows: Edwardsiella tarda, ACY83602.1; Escherichia coli str. K-12 substr, NP_418555.1; Salamae, VEA01344.1; Aeromonas veronii, QHC07268.1; Vibrio cholerae, WP_188372281.1; Klebsiella pneumoniae, AZH95435.1; Shigella boydii, PHU85906.1. The GenBank accession numbers of CadB homologues are as follows: E. tarda, ACY83601.1; Salmonella enterica, WP_000100010.1; Klebsiella pneumoniae, WP_110924908.1; Escherichia coli, WP_175119720.1; Shigella flexneri, NP_709997.1; Vibrio cholerae, WP_189017881.1; Aeromonas veronii, WP_118881724.1. [file 13567_2021_987_MOESM1_ESM.doc]

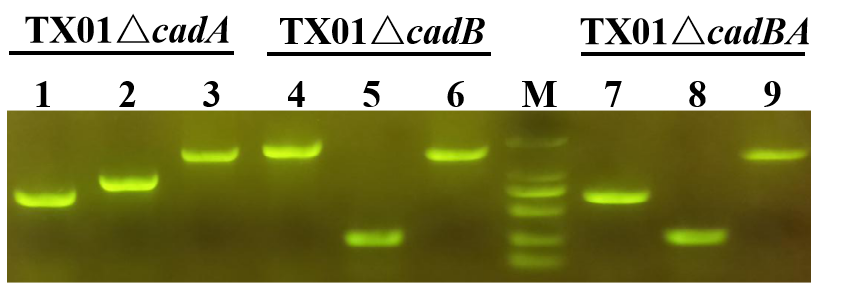

Supplement: Supplementary file 2 — Additional file 2.Validation of TX01 mutants by PCR amplification. Lanes 1 and 7 were the 661-bp (deletion of cadA allele) fragments amplified from TX01ΔcadA and TX01ΔcadBA genomic DNA with primers CadAKOF3/CadAKOR3, respectively. Lane 4 was 1684-bp cadA fragment. Lanes 5 and 8 were the 265 bp (deletion of cadB allele) fragments amplified from TX01ΔcadB and TX01ΔcadBA genomic DNA with primers CadBKOF3/CadBKOR3, respectively. Lane 2 was 901-bp cadB fragment. Lanes 3, 6 and 9 were 16S rRNA gene fragments amplified from three mutants with universal primers 27F/1492R. M is DS 2000 marker. [file 13567_2021_987_MOESM2_ESM.doc]

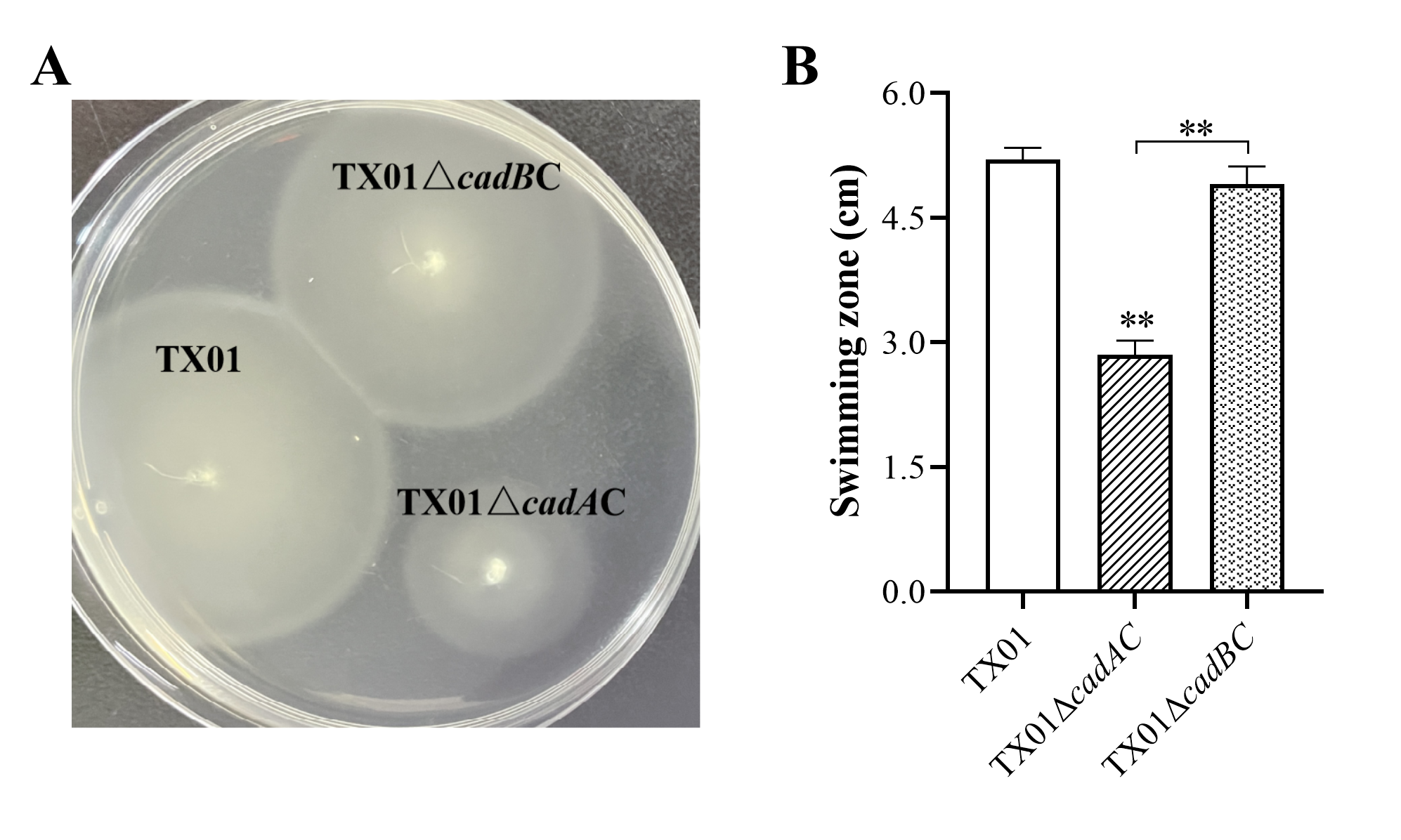

Supplement: Supplementary file 3 — Additional file 3.The swimming ability of complementary strains TX01ΔcadAC and TX01ΔcadBC in the acid swimming plate. TX01, TX01ΔcadAC, and TX01ΔcadBC were cultured in LB medium to an OD600 of 0.5, then aliquots of cell suspensions (1 μL) were inoculated into the center of swimming plates including 0.3% (W/V) agar with pH = 5.5 at 30 °C for 18 h (A). The diameter of the swimming zone from the swimming plate at pH = 5.5 (B). The data are presented as the means ± SEM (N = 3). N, the number of times the experiment was performed. **, P < 0.01. [file 13567_2021_987_MOESM3_ESM.doc]

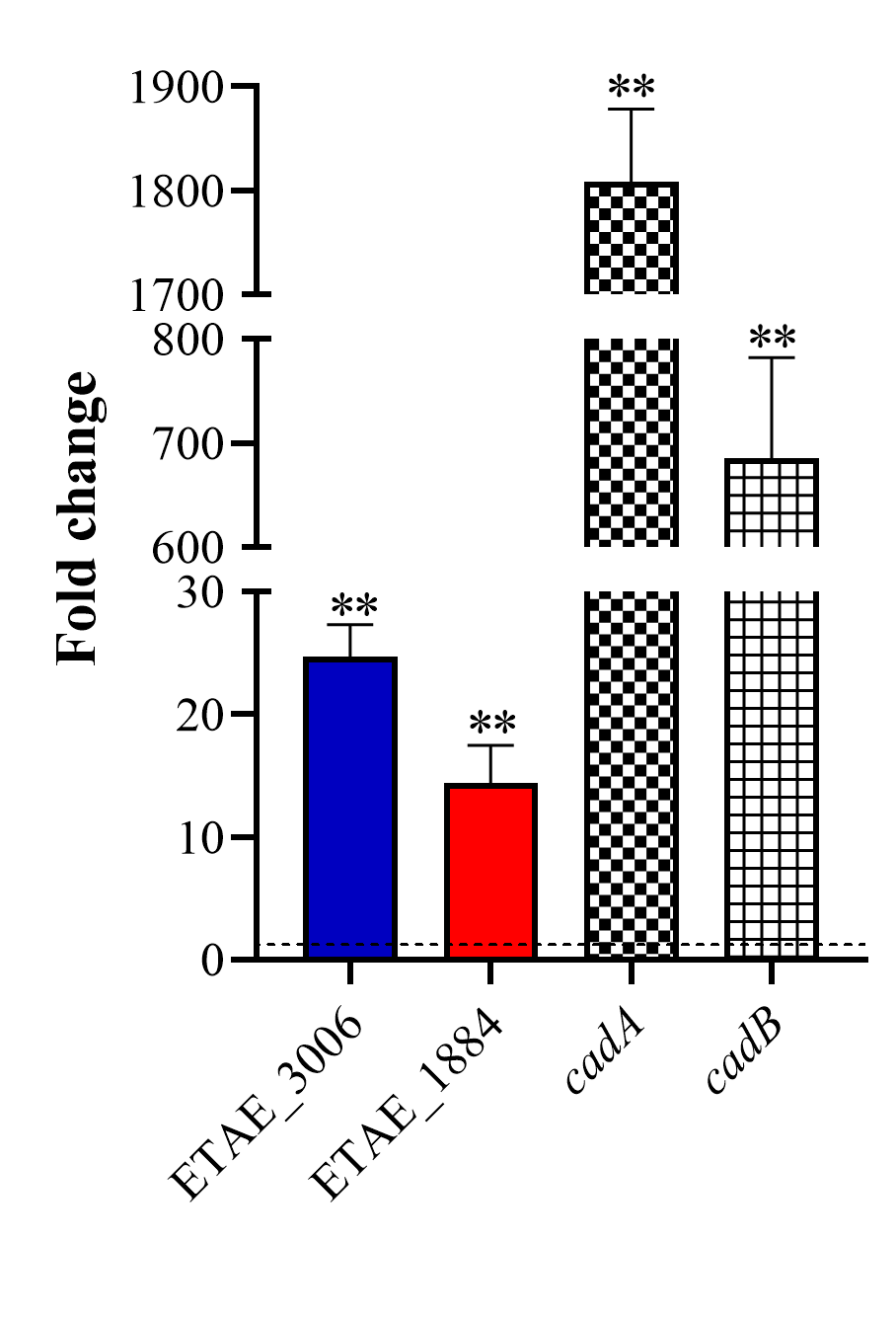

Supplement: Supplementary file 4 — Additional file 4.The expression of acid resistance genes in Edwardsiella tarda under acidic conditions. The exponential phase of overnight cultures of TX01 were grown in normal LB medium at pH 7.0 and acidic medium at pH 5.5 for 1 h. The relative expression of ETAE_3006 and ETAE_1884 were determined by RT-qPCR. The fold difference derived from the values under acidic conditions compared with the values under normal conditions. Data are presented as the means ± SEM (N = 3). N, the number of times the experiment was performed. **, P < 0.01. [file 13567_2021_987_MOESM4_ESM.docx]
